# Dissociation of retinal and headcentric disparity signals in dorsal human cortex

**DOI:** 10.3389/fnsys.2015.00016

**Published:** 2015-02-24

**Authors:** David M. Arnoldussen, Jeroen Goossens, Albert V. van Den Berg

**Affiliations:** ^1^Section Biophysics, Department of Cognitive Neuroscience, Radboud University Nijmegen Medical Centre, Donders Institute for Brain, Cognition, and BehaviorNijmegen, Netherlands; ^2^School of Psychology, University of NottinghamNottingham, UK

**Keywords:** motion-in-depth, optic flow, self-motion, stereo, vergence eye movements, headcentric disparity, retinal disparity

## Abstract

Recent fMRI studies have shown fusion of visual motion and disparity signals for shape perception (Ban et al., 2012), and unmasking camouflaged surfaces (Rokers et al., 2009), but no such interaction is known for typical dorsal motion pathway tasks, like grasping and navigation. Here, we investigate human speed perception of forward motion and its representation in the human motion network. We observe strong interaction in medial (V3ab, V6) and lateral motion areas (MT^+^), which differ significantly. Whereas the retinal disparity dominates the binocular contribution to the BOLD activity in the anterior part of area MT^+^, headcentric disparity modulation of the BOLD response dominates in area V3ab and V6. This suggests that medial motion areas not only represent rotational speed of the *head* (Arnoldussen et al., 2011), but also translational speed of the *head* relative to the scene. Interestingly, a strong response to vergence eye movements was found in area V1, which showed a dependency on visual direction, just like vertical-size disparity. This is the first report of a vertical-size disparity correlate in human striate cortex.

## Introduction

Optic flow, or the pattern of visual motion received by a moving observer, carries important information on the layout of the environment and self-motion (for reviews: Koenderink, 1986; Warren, 2004). Most of this work has dealt with the optic flow of a single vantage point, disregarding the potential contribution of binocular visual information to optic flow processing. The one-eyed pilot Wiley Post-provides an intriguing example of the common notion that binocular information is *not essential* for complex navigation tasks (Regan and Gray, 2009), but that does not imply that the binocular contribution to optic flow processing is absent. Binocular vision could improve the judgment of direction and speed of self-motion from optic flow. Indeed, various perceptual studies using a complex mixture of rotation and forward self-motion have provided evidence for this notion; e.g., stereo signals help to recover the percept of *direction* of self-motion in the presence of noise (Van den Berg and Brenner, 1994a) or to prevent a biased heading percept due to parallel motion in a transparent overlay (Grigo and Lappe, 1998). The *feeling* of self motion (vection) is also faster and stronger for self-motion consistent than for self-motion inconsistent stereo signals (Palmisano, 2002).

*Imaging* studies found *overlapping responses* to optic flow and stereoscopic signals in the dorsal visual pathway (Tsao et al., 2003; Brouwer et al., 2005; Likova and Tyler, 2007) pointing to potential interactions between binocular and flow signals. More recently, interactions specific to *joint processing* of motion and stereoscopic signals were reported (Smith and Wall, 2008; Rokers et al., 2009; Ban et al., 2012; Seymour and Clifford, 2012), however, *not* using self-motion stimuli.

The BOLD response in parietal-occipital sulcus (POS) increases with the presence of a gradient of stereoscopic depth, but only when this gradient is consistent with optic flow of forward self-motion (Cardin and Smith, 2011). Within anterior POS and the MT^+^-complex, BOLD correlates to the stereo-related increase of the vection percept to simulated forward motion were reported (Kovacs et al., 2008). Together, the latter studies suggest that medial and lateral motion areas may play a role in integration of disparity signals with optic flow for direction and/or forward speed of self-motion.

Traveled distance over time cannot be recovered solely from optic flow (Koenderink and Doorn, 1987). Yet, humans are quite accurate at such judgments when optic flow is augmented with stereo signals (Campos et al., 2012). Could binocular signals somehow provide a distance scale to enable distance judgments? By itself, the change in retinal disparity of an object that accompanies self-motion cannot provide this, because retinal disparity depends on the object's depth relative to fixation not on egocentric distance. By contrast, an object's headcentric disparity (H) provides egocentric distance which could provide the missing information. Headcentric disparity of an object is defined as the difference angle between the brain's representation of the two eyes' headcentric directions toward that object (Erkelens and van Ee, 1998). That theoretical study offered an interesting analysis how the vertical component of headcentric disparity could allow the visual system to register and correct errors in signaled eye-in-head posture, while the horizontal component of headcentric disparity would reveal the distances in the visual scene. Because headcentric direction of each eye is not given of the retinal image alone, the notion implies convergence of retinal signals with extra-retinal sources like efference copy or proprioceptive signals about the eyes' orientations in the head. To our knowledge there is no physiological evidence for the concept of headcentric disparity but a recent behavioral study intriguingly reports depth percepts consistent with non-zero headcentric disparity just prior to a saccade when retinal disparity is still zero (Zhang et al., 2010). Here we study percepts and hemodynamic signals that distinguish between changes of retinal and headcentric disparity over time.

We report psychophysical evidence that motion in depth, perceived from identical optic flow, increases in proportion to the amount of changing headcentric disparity. BOLD responses in medial motion areas also increased in proportion to the added headcentric disparity. On the other hand, the anterior part of the MT^+^-complex responded to the optic flow and retinal disparity amplitude. These results add to accumulating evidence that medial and lateral motion areas contribute distinctly to self-motion perception.

## Materials and methods

### Subjects

Eight healthy subjects participated in the fMRI experiment (3 female). Four of them participated in the perceptual study (1 female); five of them participated in the vergence eye movement measurements (2 female). All subjects passed a stereo-acuity test (TNO, Netherlands Scientific Organization). We obtained written informed consent from all subjects prior to scanning, and procedures were approved by the Radboud University Medical Centre. Five subjects were experienced with optic flow stimuli and participated previously in other fMRI studies. All subjects but one (one of the authors) were naïve to the purpose of the study.

### Disentangling binocular cues to self-motion

Natural self-motion in depth offers simultaneous modulation of 3D motion, retinal disparity, and headcentric disparity. To assess their individual contributions to the self-motion percept and the evoked BOLD signals, we developed a novel stimulus that allowed full 3D stereo flow presentation with preserved motion parallax, while retinal disparity-, headcentric disparity- and vergence amplitude could be varied independently.

**Figure 1:**
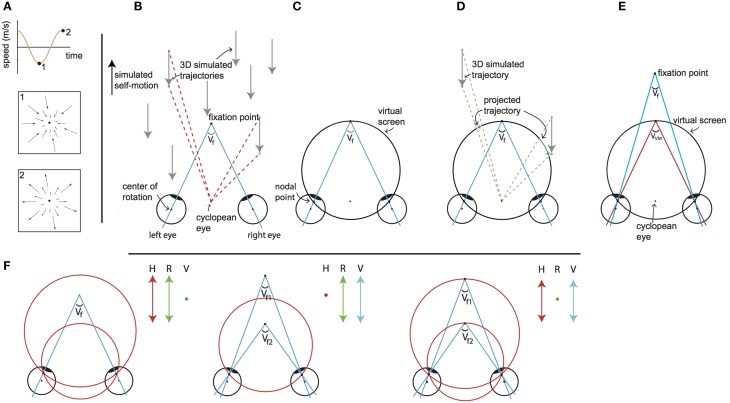
**Visual stimulus explanation and rationale**. We equated disparity for all elements in the scene by projection of the 3D cloud on the VM torus, for every element for every frame. The objects on this “virtual projection screen” are in the final stage of each motion frame projected on the real screen in the scanner (not depicted). Seen from the cyclopean eye, 3D motion does not change by the intermediate projection step onto the virtual screen, while that intermediate projection makes horizontal retinal disparity nearly constant for all points. Thus, we decouple the motion field of the cyclopean eye from the binocular disparity. By changing the simulated distances of fixation point and the target vergence of the VM surface independently, one decouples changes of retinal disparity and headcentric disparity. This is illustrated in detail in the following panels. **(A)** The visual stimulus consists of an optic flow pattern that simulates oscillating (forward/backward) self-motion in depth. The pictograms show the point-motion vectors when the simulated backward (1) and forward (2) speed was maximal. At the zero crossings, simulated self-motion speed was zero and all points were stationary at that instance. **(B)** Top view of simulated forward motion (black arrow on the left), by presenting approaching dots (gray arrows). For two dots the angular displacement as seen from the cyclopean eye is illustrated (red dashed lines), while the eyes are converged with an angle *V_f_*. Note that the angular velocities and the changing disparities are different for the two points. **(C)** The top view shows a cross section of the virtual screen, which consists of the Vieth-Muller circle that runs through the fixation point and the eyes' nodal points. The virtual screen is a surface of revolution of the Vieth-Muller circle about the inter-ocular axis. Across the virtual screen the vergence angle of the eyes required to fixate that point (target vergence: TV) is identical. Hence the virtual screen is an iso-vergence surface. **(D)** To disentangle retinal and headcentric disparity the point motions in **(B)** are projected on the virtual screen. The projected trajectories as seen from the cyclopean eye are identical to those illustrated by the red dashed lines in **(B)**, showing that the flow as seen from the cyclopean eye remains. However, because the dots are now moving across the virtual screen their TV is constant, which holds for all points on the surface. For the illustrated cross section of the virtual screen, the points' *retinal* disparities are zero throughout the trajectory. This is not exactly true for points below and above, but the deviations from zero are small for elevations of less than ±25°. **(E)** When the fixation point moves in depth dissociating the vergence angle of the eyes (*V_f_*) and the target vergence of the virtual screen (V_VM_) the retinal horizontal disparities of the points that are projected on the virtual surface increase by the difference of those vergence angles (with small deviations depending on the elevation). **(F)** Our paradigm (to a good approximation) dissociates horizontal retinal disparity and headcentric disparity. The *left* panel illustrates co-variation of retinal (green arrow) and headcentric disparity (red arrow) while vergence is fixed (blue dot). This happens, when the virtual screen grows to recede in the distance and shrinks on approach. The *middle* panel illustrates how retinal disparity and eye vergence (blue arrow) co-vary while headcentric disparity remains constant (red dot). This happens, when the eyes converge and diverge in pursuit of the fixation point's motion in depth, while the virtual screen is fixed. The *right* panel illustrates how headcentric disparity and eye vergence co-vary while retinal disparity is virtually constant (green dot), as occurs when the fixation point remains located in the virtual screen while it grows and shrinks.

Our stimulus started out with simulation of self-motion on a linear track with a sinusoidal speed as a function of time (Figure 1A). Seen from the front on the screen, this resulted in typical self-motion associated flow patterns, alternating expansion flow (forward motion) and contraction flow (backward motion, Figure 1A bottom panels).

Figure 1B illustrates a top view of an observer's converged eyes, during a forward self-motion that was simulated by a collection of approaching point targets (gray arrows). Red dashed lines illustrate the motion trajectory of different flow points as seen from the cyclopean eye, for a given time lapse. The apparent angle difference between the two flow points illustrates motion parallax, which signals monocular self-motion in depth. The motion parallax naturally is concomitant with different changes in disparity. Our goal was to maintain motion parallax, but remove differences in disparity.

This was achieved by using a virtual screen (Figure 1C), on which all flow were projected (Figure 1D). The virtual screen has the specific property that fixation on any point on its surface requires the same eye vergence. Seen on top (Figure 1C), it describes a circle (a Vieth-Muller (VM) circle), connecting one target point in front of the head and the centers of both eyes. In 3D, the virtual screen has the shape of a toroidal surface—the VM surface—which is the extension of the Vieth-Muller circle to a surface, by rotation of that circle about the inter-ocular axis. It can be thought of as an oculomotor measure of egocentric distance. Hence, after projection of the flow points on the screen, required vergence angle for fixation on any point was equated (Figure 1D).

The center of projection was the cyclopean eye, located midway between the subject's eyes (Figure 1D). Each projected point on the VM surface was presented at a distance “d” from the cyclopean eye:

(1) $$\text{d}=\frac{1}{2}\text{ IOD}\left(\text{cos}(\alpha )\text{cot}(V_{\text{VM}})+\sqrt{{\text{csc}}^{2}(V_{\text{VM}})-{\text{cot}}^{2}(V_{\text{VM}}){\text{sin}}^{2}(\alpha )}\right)$$

here IOD denotes the interocular distance and *α* the azimuthal direction of the point.

We define the required eye vergence to fixate a point (irrespective of the current eye vergence) as the *target vergence* (TV) of that point. As a result of the projection on the VM surface, all projected points maintain throughout their motion a constant TV that equals the target vergence level of the VM surface (V_VM_). Note that V_VM_ and the azimuth angle determine the distance from the cyclopean eye (d) of the point that is projected on the virtual screen as defined in Equation (1).

Naturally, the three dimensional positions of points on the VM surface are given in Helmholtz angles: azimuth (α) and elevation (θ) and *V_VM_*. For fixation in the horizontal plane (i.e., without cyclovergence of the eyes), the horizontal *retinal disparity* of points on the VM surface is close to zero. It is *exactly* zero for points on a vertical cylinder through the Vieth-Muller circle (Howard and Rogers, 2002). Points of the fixated VM surface that are elevated above or below the fixation point therefore possess crossed horizontal disparities, which turned out to be small, especially when the extent of the display was limited to ±25° of elevation (see Supplementary Material—Methods).

In some conditions we also simulated motion in depth of the (dichoptic) fixation ring, evoking eye vergence. Then, the difference of the target vergence of the fixation point (*V_f_*) and the target vergence of the VM surface (V_VM_, Figure 1E) determines the *horizontal retinal disparity*. Changes in horizontal retinal disparity by moving the fixation point out of the VM surface do not *exactly* generate horizontal retinal disparity (*V_f_* - *V_VM_*) across the entire VM surface, but spatially averaged deviations from this approximation are small (see Supplementary Material—Methods).

Finally, *headcentric disparity* of a point is defined as the vergence angle that would be required to fixate that point, irrespective of the current eye vergence. Hence, headcentric disparity of a stimulus point equals its TV. We prefer to use this term in the remainder of the article to stress its perceptual attribute and its connection to the retinal disparity.

The levels of retinal and headcentric disparity could be varied independently by moving the fixation point in depth relative to the VM surface and by shrinking or growing the size of the VM torus.

This way, we presented an optic flow pattern (as seen from the ego-center) that was fully consistent with a sinusoidal movement in depth including motion parallax cues to depth. Yet, for all points headcentric disparity was constant across the visual field, and horizontal retinocentric disparity was approximately constant. Both could be varied independently over time, by changing the target vergence angles of the virtual projection screen (V_VM_, Figure 1F left), the fixation ring (*V_f_*, Figure 1F middle), or both (Figure 1F right).

The flow speed was varied sinusoidally (frequency: 1/6 Hz) over time (Figure 1A). The parameters of the flow (see below) were identical for *all* conditions. Just like the flow speed, the distance of the fixation point and the VM surface varied sinusoidally (1/6 Hz), always starting at the same target vergence of −6.5°.

**Figure 2:**
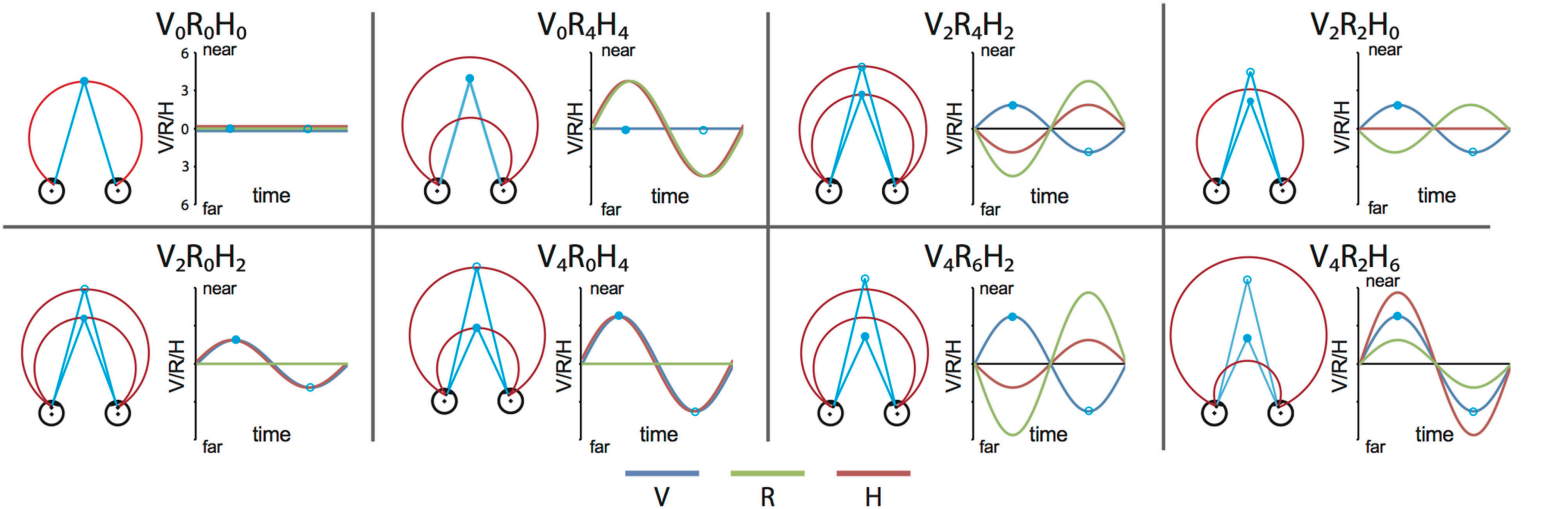
**Stimulus conditions**. Each panel shows one stimulus condition. The right side of each box illustrates the sinusoidal variation of the binocular variables during one period, for V, R, and H (blue, green, and red line respectively). Optic flow was present in all conditions. Note that peak velocity of the flow coincides with zero crossing of V, R, and H (Figure 1A). The left side of each box shows a top view of the eyes, together with the extreme simulated positions (phase angles: 0.5 π (filled blue dot) and 1.5 π (blue circle) of the Vieth-Muller torus (red circle segments), and the eye fixation target (blue dot, blue circle). Note that in panels V_2_R_4_H_2_ and V_4_R_6_H_2_ the movements of the VM surface and the fixation point are opposite at all times.

**Figure 3:**
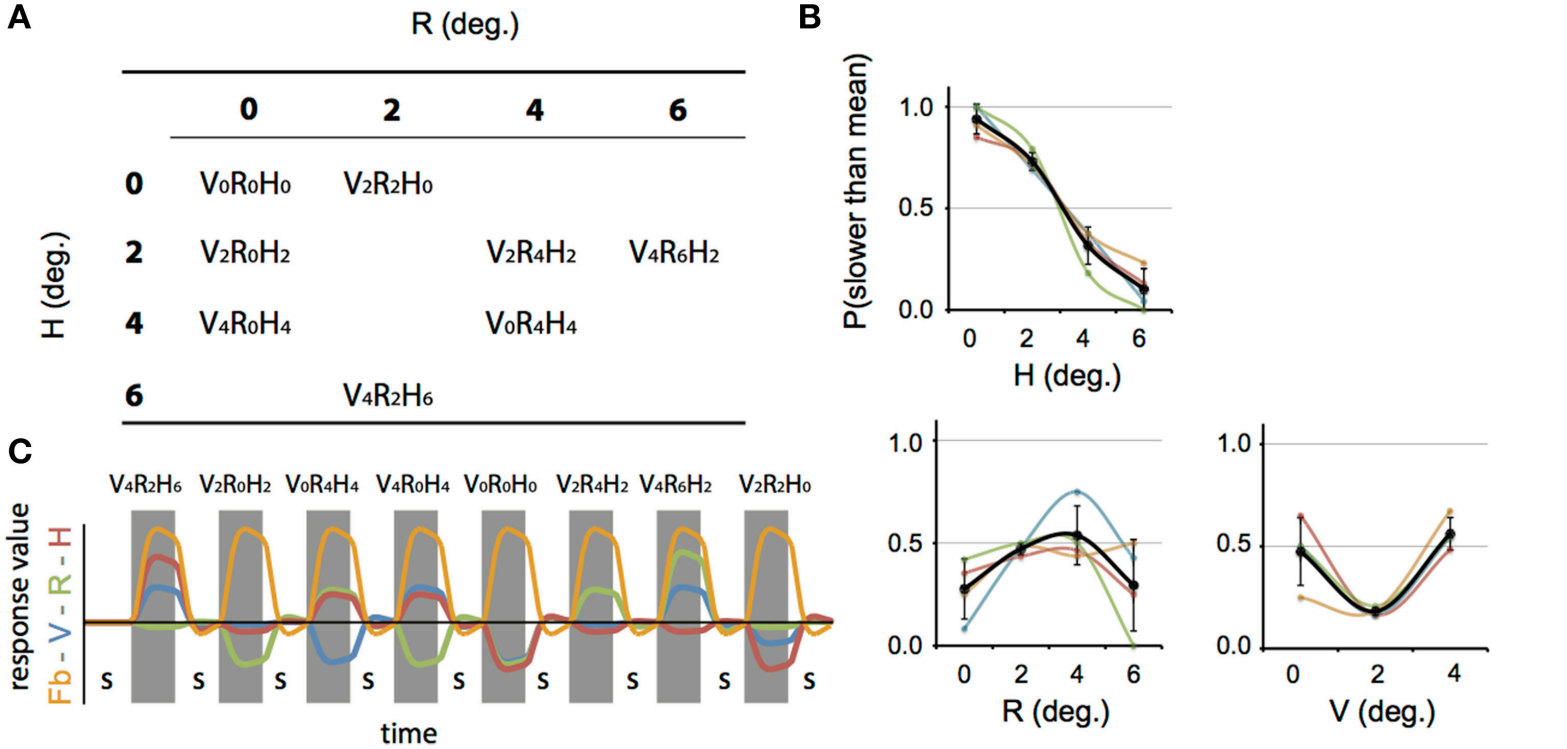
**Stimulus conditions, results perceptual study, and parametric GLM model. (A)** Table of the 8 stimulus conditions, ordered by R and H amplitude. The combination of conditions allowed for a dissociation between contributions of V, R, and H to depth perception and the BOLD modulation. **(B)** Plotted are the average proportion of “slower” responses against the amplitude of H, R, and V (bold black line in left, middle, and right graph respectively). Individual results are drawn in colors. A clear monotonic decrease of slower responses occurs for increasing levels of H amplitude, but not for R and V. **(C)** Illustration of the time courses for the four experimental predictors of the 4P-model (confound predictors not shown), convoluted with the hemodynamic response function, used in the GLM analysis. In each run (156 volumes), all 8 conditions were presented, interleaved with a baseline condition (S, random static dot pattern). The Fb predictor captures modulation of the flow (orange). In addition, three parametric predictors (scaled with respect to their amplitude) fit modulations of the BOLD signal that are linearly related to changes in V (blue), R (green), or H amplitude (red). The parametric predictors are de-meaned to approximately orthogonalize the parametric predictors with one another.

We constructed a set of 8 stimulus conditions, in which we combined three amplitudes of eye vergence (V: 0, 2, and 4° of TV of the fixation ring) with 4 headcentric disparity amplitudes (H: 0, 2, 4, and 6° of TV of the virtual screen), which resulted in 4 amplitudes of retinocentric disparity (R: 0, 2, 4, 6°; Figures 2, 3A). Notice that we describe the amplitude of V, R, and H as the amplitude of the sinusoidal displacement. Subjects were instructed to rate the self-*motion* percept based on the velocity of the motion relative to their head (see below). Because we used a fixed frequency of 1/6 Hz, amplitude and peak velocity had a fixed ratio that was very close to 1. Labeling the levels of the stimuli by peak velocity or amplitude was therefore considered equivalent (Figures 2, 3A).

### Stimulus presentation

We used a custom-built wide-field visual projection system, previously described in more detail (Arnoldussen et al., 2011). Briefly, we projected the visual stimulus on a small projection screen very close to the subject's eyes (~3 cm). Subjects wore eye contact lenses (+25 to +33 D, dependent on individual refraction errors) in both eyes to allow for easy accommodation at this distance. After positioning into the head coil, the screen distance was adjusted until the subject reported sharp viewing for both eyes on the screen. Clay putty was put on the nose bridge to prevent cross viewing of the left and right eye projections. Next, an eye calibration procedure was performed inside the bore to assess the precise position of the eyes relative to the stimulus screen (Van den Berg, 1996). To check the calibration, a fixation mark was projected in stereo on a simulated distance of 5 meters. If the subject did not report easy fusion of the fixation spot in depth, the calibration procedure was repeated. Then, some of the stimulus conditions were shown prior to scanning to familiarize the subject with pursuit in depth. We ensured that the subject reported seeing the fixation point at eye level and reported the binocular fixation point moved in depth according to the subject's straight ahead.

### Visual stimulus

All stimuli were generated on a Macintosh MacBook Pro Notebook, using openGL-rendering software. The same set of stimulus conditions was used for the psychophysical and the imaging studies. The simulated scene was a 3D stereoscopically presented cloud of dots (250 white dots against a black background) with one fixation ring. Dots were asynchronously refreshed every 1.0 s and distributed randomly within a volume in front of the eyes. Volume dimensions were 8600 (H) × 6000 (V) × 2500 (D) mm, with the nearest surface abutting the projection screen (0.75 × distance of the actual screen or about 2.3 cm). Pixel-size of the projection on the screen was 0.2–0.34°., depending on eccentricity (large at the center because of the nearer distance, smallest at largest eccentricity of 60°). Because we applied sub-pixel positioning by OpenGL software, 1/10 the of the pixel displacement resolution is offered, i.e., 0.02–0.034°. Oscillation frequency (f) of 1/6 Hz, dot- displacement amplitude (287 mm) and peak speed of dot movement (A_Flow_ = 300 mm/s) were fixed for all stimulus conditions.

In some conditions we also simulated motion in depth of the (dichoptic) fixation ring, evoking eye vergence. We note that the accommodation demand during the eye vergence conditions was smaller and opposite from normal viewing. Convergence was concomitant with *increased* rather than *decreased* distance between eye and the fixated position on the screen. However, that distance was nearly constant (about 1% variation during the largest simulated depth excursions of the fixation target).

### Perceptual study

In a psychophysics session, subjects (*n* = 5, all of whom participated in the fMRI experiment) were seated upright and with the head stabilized by a bite-board, viewing the screen at approximately the same distance as during the scanning session (~3 cm). Procedures and setup were similar to the fMRI setup described below. Subjects viewed all stimulus conditions in random order without static conditions and for a shorter period than in the fMRI experiment (*T* = 2 s). Subjects were instructed to judge for each trial the speed at which the scene was oscillating in depth relative to their head. Subjects pressed keyboard buttons, to indicate the speed as slower or faster than an internal mean, which they built up during the experiment.

Because V, R, and H amplitudes were distributed unequally over the 8 stimulus conditions (Figure 3A), the number of repetitions for V, R, and H amplitudes could not all be balanced. We chose to balance the number of repetitions across different levels of H amplitude, offering an unequal amount of repetitions for each stimulus condition. This allows us to determine the psychometric function for each level of H with equal reliability but not so for R and V. Irrespective of that reliability we expect a monotonic relation between at most one of the predictors' stimulus level and “slower” choice-frequency because the levels can be arranged as an ascending ordered set only for one predictor at a time.

### Visual stimuli—functional localizers

Closely following our procedures in a previous study (Arnoldussen et al., 2011) we identified the main visual regions V1, V3ab, and V6 through wide-field retinotopy (Pitzalis et al., 2006), and distinguished sub-regions within the flow-responsive posterior portion of the medial inferior temporal sulcus as described before (MT^+^; Dukelow et al., 2001; Huk et al., 2002). We distinguished MT^+^/contralateral (MT^+^/c), which responds only to flow presented in the contra-lateral hemifield, and MT^+^/bilateral (MT^+/^b) which responds also to flow presented in the ipsi-lateral hemifield (MT^+^/bilateral, (MT^+^/b). We refer to the MT^+^ sub-regions in this way, because of growing evidence that they likely constitute several different functional regions (Kolster et al., 2010). The admittedly crude dissection of MT^+^ in bi-lateral and contra-lateral flow-sensitive sub-regions is useful nonetheless; a bi-laterally responsive sub-region is likely to have a distinctive functional contribution to wide-field flow processing and may show a different type of integration with binocular cues.

### Design fMRI

The 8 stimulus conditions were presented in blocks of 18 s (3 periods), interleaved by 18 s of the baseline condition, resulting in runs of about 5 min. The conditions were presented pseudo-randomly and counter-balanced. The baseline condition was a pattern of stationary dots, placed randomly and independently for each eye (i.e., no binocular matches) with limited lifetime (1.0 s). Subjects were instructed to fixate the binocular fixation ring with TV angle of −6.5° during the static condition and to follow the binocular target when it moved in depth. Subjects performed 8 (subject 7 and 8) to 16 (subject 1 and 3) runs in total (average: 12.8 runs). All fMRI acquisition took place in three (subject 1 and 3) or two separate sessions.

### MRI acquisition

The MRI experiment was conducted on a 3 Tesla TIM trio Siemens scanner at the Donders Center for Cognitive Neuro-imaging (Nijmegen, The Netherlands). For each subject, we obtained a high-resolution full-brain anatomical scan using a 32-channel head coil (T1-weighted MPRAGE, 192 slices, 256 × 256 matrix, resolution of 1 mm^3^).

For the experimental scans, we used an 8-channel occipital surface coil. Scans were obtained with a resolution of 2 mm iso-voxel [T2^*^-weighted; single-shot echo-planar imaging; 32 slices; repetition time (TR): 2 s; echo time (TE): 30 ms]. The experimental runs consisted of 156 volume acquisitions. The initial 2 TR's were discarded to account for scanner drifts.

Subjects also performed 6 localizer runs (2 for polar angle, 2 for eccentricity, 2 for MT^+^/b-MT^+^/c localizer).

### Preprocessing fMRI data

Brainvoyager QX (BVQX, version 2.6) was used for the analysis of functional and anatomical images (Brain Innovation, Maastricht, The Netherlands). All functional runs were corrected for motion and inter-slice scan time differences and were subsequently aligned with the high-resolution anatomical scan. For the retinotopy and MT/MST localizer scans, a high-pass filter was applied on each voxel's data time course by removing the fit of a General Linear Model (GLM) with predictors defined by a linear, a sine and a cosine function (the latter two with a period equal to the run duration). For the experimental runs, these three predictors were added as confound predictors to the GLM model, both for individual and multi-subject GLM analyses.

Using the high-resolution anatomical scan, white and gray matter boundaries were defined and used for the construction of the inflated and flattened representation of each subject's left and right hemisphere (using automatic segmentation by BVQX, followed by manual refinement).

### Analysis—functional localizers

The phase of the BOLD response to the retinotopic stimuli (rotating wedge, expanding ring) was determined by cross-correlation. Boundaries of visual areas V1 and V3ab were defined based on the phase reversal of the polar angle mapping stimulus by eye, on the flattened representation of each subject's left and right hemisphere (Sereno et al., 1995). In only a few hemispheres, we could distinguish the boundary between v3b and v3a on the basis of the retinotopy (Larsson and Heeger, 2006). Therefore, we decided to treat these regions as a single ROI, which we refer to as V3ab. V6 was defined as a region within the POS, medial to V3ab, containing an entire representation of the contra-lateral hemifield and an eccentricity map (Pitzalis et al., 2006).

For the MT^+^/b-MT^+^/c localizer, we used a GLM with separate predictors for full field, left-visual-field, and right-visual-field flow presentation. First, the region within the medial temporal sulcus that was highly responsive (*p* < 0.01, uncorrected) to a GLM contrast between full field optic flow and the static conditions was identified as MT^+^. MT^+^/b was then defined as an anterior sub-region of MT^+^ containing all contiguous voxels that responded to both ipsi-lateral and contra-lateral flow (with respect to the current hemipshere, *p* < 0. 05 uncorrected; Dukelow et al., 2001; Huk et al., 2002). MT^+^/c was defined as a posterior sub-region of MT^+^ that consisted of all contiguous voxels responsive to contra-lateral flow (*p* < 0.01 uncorrected).

### fMRI analysis

For the searchlight approach and subsequent multi-subject analyses, we used a parametric GLM model with 4 main predictors (4P-model; Figure 3C), described by:

(2) $$\begin{array}{l} Y=\beta_{1}{}^{*}Fb+\beta_{2}{}^{*}V+\beta_{3}{}^{*}R+\beta_{4}{}^{*}H\\ \;+\beta_{5}{}^{*}L+\beta_{6}{}^{*}S+\beta_{7}{}^{*}C+\beta_{8} \end{array}$$

The first predictor captures the general BOLD response to the flow (Fb-predictor), which was identical for all conditions. The next three predictors capture parametric modulations of the BOLD signal by the amplitude of vergence (V-predictor), retinal disparity (R-predictor), and headcentric disparity (H-predictor; Figure 3C). Note that the β-values for the parametric predictors do not reflect level activation, but rather a region's linear sensitivity to amplitude changes in V, R, or H. β-values thus indicate % BOLD-signal per degree amplitude.

Predictor-values for the different conditions were offset to a mean of zero (Wood et al., 2008) to improve the orthogonality of the predictor functions. We included three confound predictors (a linear trend (L), a sinus (S), a cosinus (C), and a baseline predictor (β_8_).

**Figure 4:**
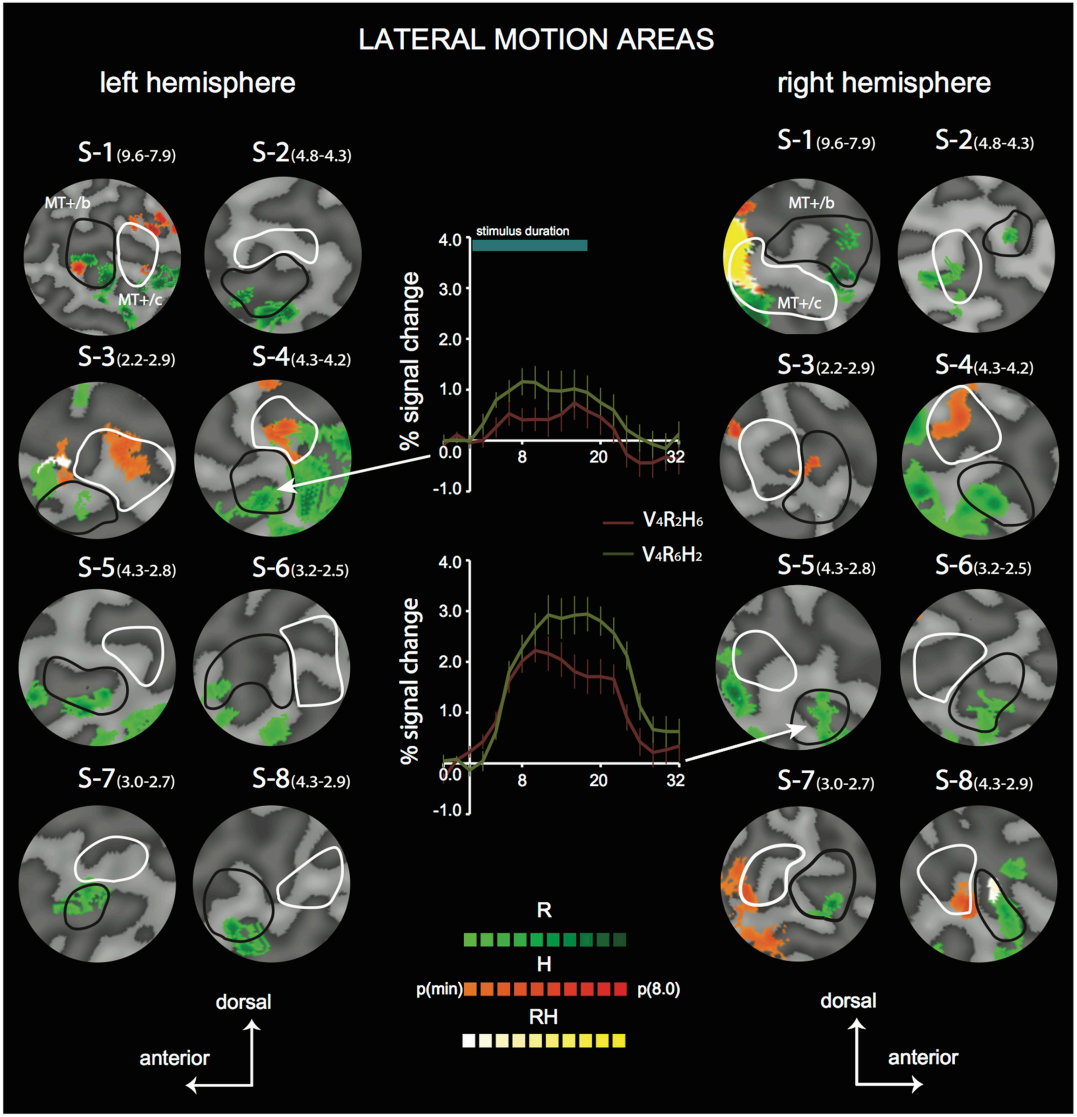
**Differential sensitivity to binocular signals in lateral motion areas**. Sections of the flattened representation of all hemispheres, encompassing MT^+^/c (white outline) and MT^+^/b (black outline). On top, peak activations are drawn for R (green—dark green), H (orange—red), and RH (white—yellow). P(min) was set independently for each hemisphere to 75% of the *p*-value at which the most significant area of activation surfaced P(t)_(min)_ values are given in parentheses following subject name. For example, P(t)_(min)_ for S-1 are 9.6 for R contrast, and 7.9 for H contrast. Center graphs show the average BOLD response for two illustrative conditions (V_4_R_2_H_6_ and V_4_R_6_H_2_), in the R peak activation area indicated by the white arrows for S4-LH (upper graph) and S5-RH (lower graph). Error bars represent SEM.

**Figure 5:**
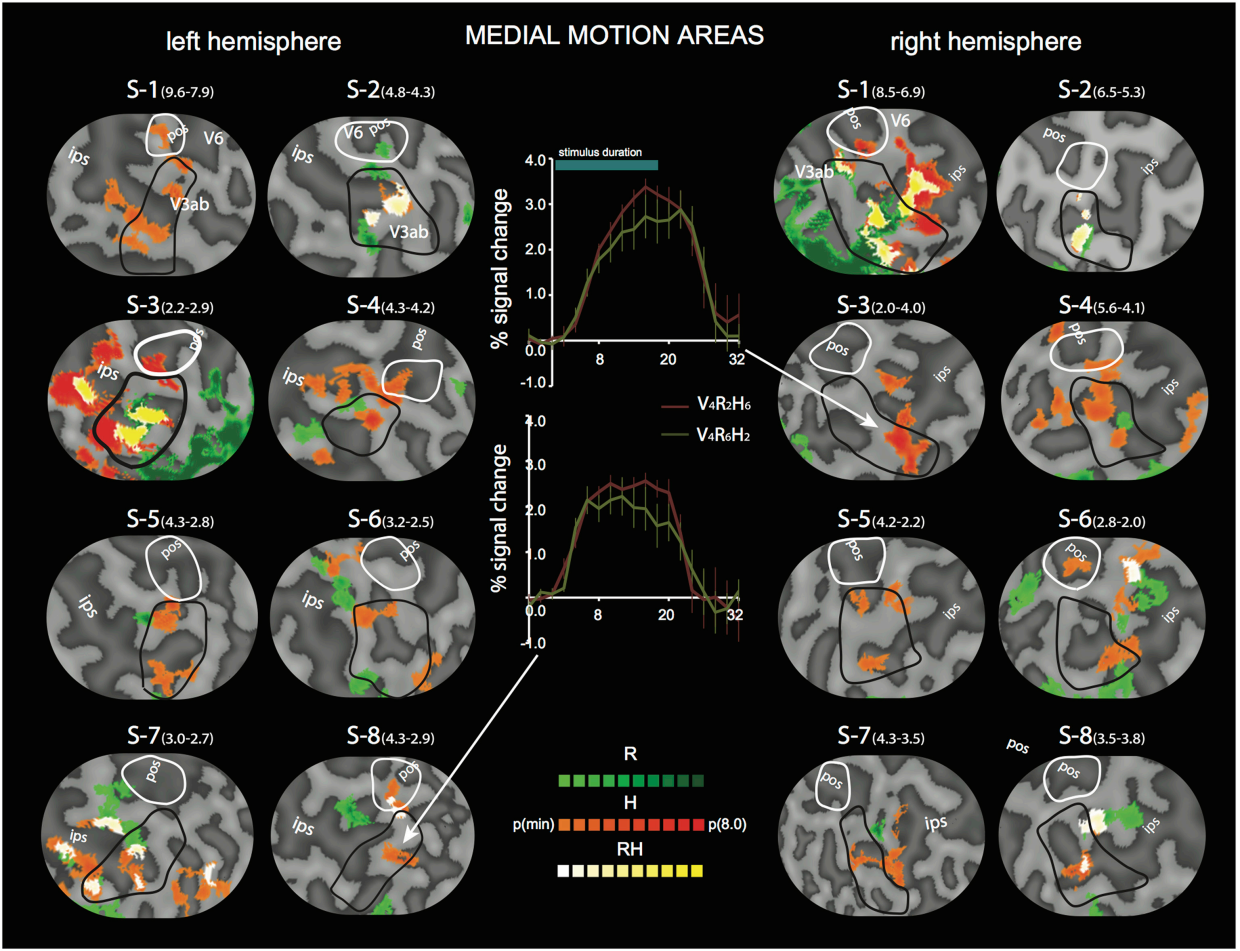
**Differential sensitivity to binocular signals in medial motion areas**. Sections of the flattened representation of all hemispheres, encompassing V6 (white outline) and V3ab (black outline). On top, peak activations are drawn for R (green—dark green). H (orange—red), and RH (white—yellow). P(t)_(min)_ was set independently for each hemisphere to 75% of the *p*-value at which the most significant area of activation surfaced. P(t)_(min)_ values are given in parentheses following subject name. Center graphs show the average BOLD response for two illustrative conditions (V_4_R_2_H_6_ and V_4_R_6_H_2_), in the H peak activation area indicated by the white arrows for S3-RH (upper graph) and S8-LH (lower graph). Error bars represent SEM.

We applied the 4P-model on the data of every hemisphere separately, without voxel restrictions. We tested for a significant difference between baseline response (dichoptic static dots) and several joint responses: Flow baseline and R (R), flow baseline and H (H), and flow baseline and R and H (RH). A conjunction analysis refers to a logical AND operation for combinations of GLM contrasts. For every hemisphere, the results for R, H, and RH are plotted on a flat map representation, as depicted in Figures 4, 5. Per hemisphere, minimum p-level for each contrast was set to 75% of the p-level at which no more blobs were visible within any of the ROIs (except V1) with a cluster threshold of 25 mm^2^. If this value was lower than *p* < 0.05, it was set to *p* < 0.05. To prevent too restrictive statistics, minimum p(t)-levels beyond p(6.0) were set to p(6.0) (equal to *p* < 2.7 e^−9^, uncorrected). P(t)_*min*_ values for each individual contrast are given in parentheses following subject name for R and H contrast, respectively.

**Figure 8B** shows the result of a GLM contrast between a conjunction of Fb and V (V), and baseline, for two hemispheres.

**Figure 6:**
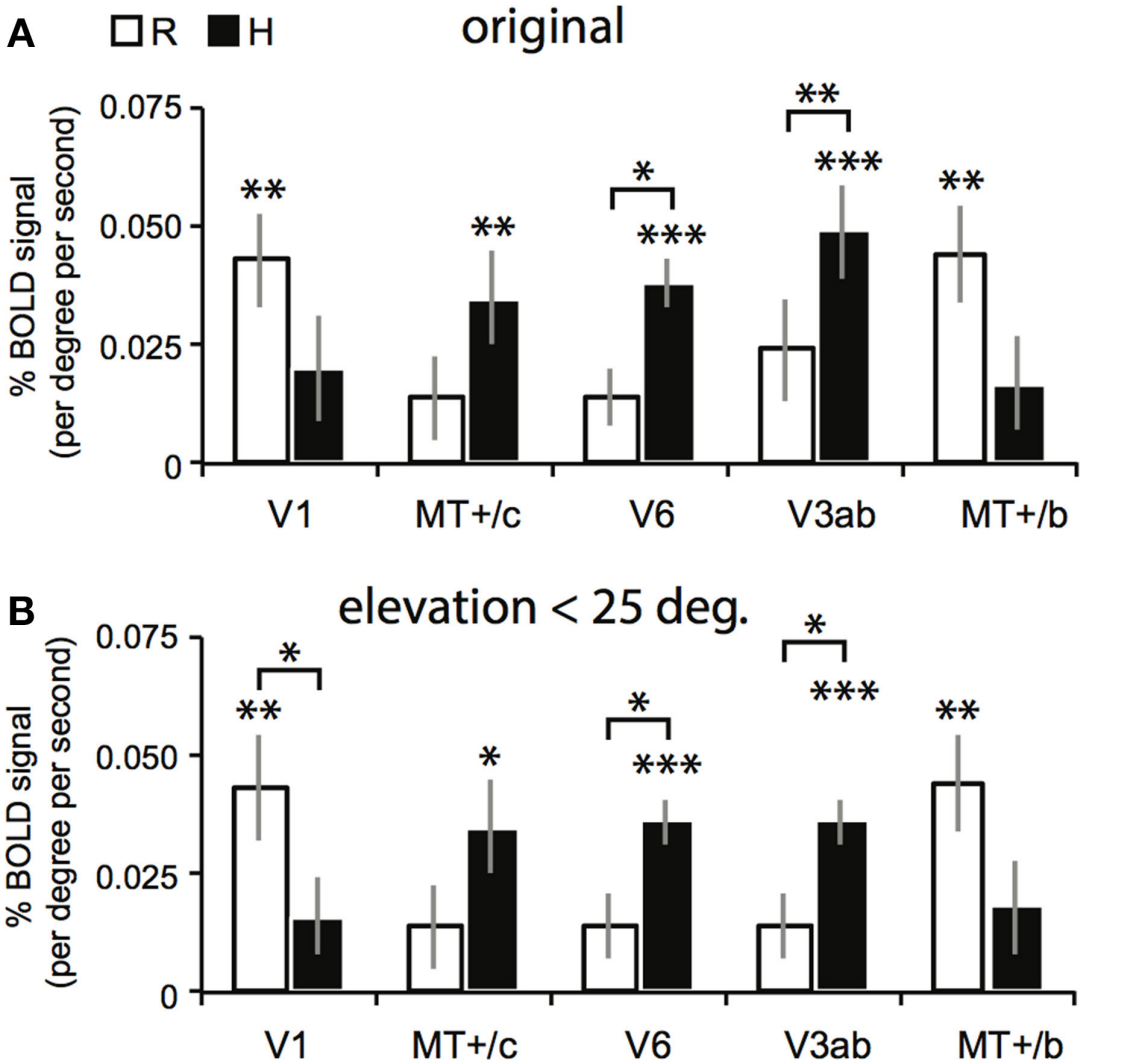
**Results for the multi-subject ROI-GLM**. **(A)** Bars show R (white) and H (black) sensitivity across subjects. V1 and MT^+^/b are significantly modulated by R [two-sided *t*-tests, *t*_(7)_ = 4.25; 4.46, ^**^p < 0.01]. MT^+^/c, V6, and V3ab are significantly modulated by H [MT^+^/c: *t*_(7)_ = 3.52, ^**^p < 0.01, V3ab, V6: *t*_(7)_ = 5.08, 7.15 ^***^p < 0.001]. Sensitivity to H was stronger than R in V6, and V3ab [*t*_(7)_ = 3.41; 3.15, V6: ^*^p < 0.05 V3ab: ^**^p < 0.01; other ROIs: *t*_(7)_ < 1.95, *p* > 0.05]. **(B)** We repeated the analysis in **(A)** with a subgroup of voxels (~90% for each ROI), whose receptive field was located within 25° of elevation in the visual field. Re-analysis on this voxel selection revealed minimal differences for R, H, and V sensitivity (V not shown) with the results shown in **(A)**. Hence, we can safely conclude that changes in retinal disparity caused by the different geometries of the isovergence shape (a torus) and the zero horizontal retinal disparity shape (a cylinder) cannot explain these results.

The multi-subject ROI results (Figures 6, **8A**) were obtained by a random effects analysis on the averaged data of each ROI, using a multi-subject GLM with predictors separated for each subject. GLM contrasts were specified for each subject and the resulting mean values across subjects was tested for significant deviation from zero (two-sided *t*-test, *p* < 0.05, uncorrected). Contrasts tested include a deviation from zero for beta values of V, R, and H separately (Figure 6 for R and H, **Figure 8A** for V); no conjunctions with the Fb predictor were included in these analyses.

We also analyzed the BOLD responses to each particular combination of binocular signals in our stimuli, using a GLM model with 8 main predictors (the 8P-model):

(3) $$Y=\sum_{i\;=\;1}^{8}\beta_{i}{}^{*}E_{i}+\beta_{9}{}^{*}L+\beta_{10}{}^{*}S+\beta_{11}{}^{*}C+\beta_{12}$$

Including a separate predictor for each experimental condition (E_1_–E_8_), three confound predictors [a Linear trend (L), a Sine (S), Cosine (C)], and baseline activity (β_12_). This allowed us for example to distinguish the condition without temporal modulation of the binocular signals (V_0_R_0_H_0_) from all the other conditions that add at least one temporally modulated binocular signal to the flow.

#### Vertical disparity—analysis

We observed in V1 a strong BOLD modulation by V, which was distributed unequally across the cortical surface (**Figure 8B**). We hypothesized that this activation is caused by the changing vertical retinal disparity during vergence eye movements.

Vertical disparity (*χ*) depends on visual direction [Helmholtz angles azimuth (*α*) and elevation (θ)] and the convergence of the eyes (TV of the fixation point: *V_f_*). This is captured by the following formula:

(4) $$\chi (\alpha ,\theta ,V_{f})=\frac{1}{2}\sin(2\theta )*\tan(\alpha ){}^{*}V_{f}$$

This formula is based on Equation (1) of Read et al. (2009) with the constraints that the mean and difference of the eyes' torsions, horizontal cyclopean eye orientation and the vertical vergence are zero (Read et al., 2009). This is appropriate for the experiments that we conducted because subjects only made slow vergence eye movement along a trajectory straight ahead at eye height.

We selected a sub-group of all V1 voxels for this analysis, i.e., those voxels that showed significance (*p* < 0.05, uncorrected) on the polar angle mapping and the eccentricity mapping, and on the GLM contrast of β_*v*_ against static baseline. (For the polar angle mapping and eccentricity mapping, significance defines a significant correlation between the voxel's time course and the optimal lag value.) We converted each voxel's polar angle (μ) and eccentricity (ecc) fit from retinotopic mapping to the corresponding azimuth and elevation angles (see Supplementary Material—Methods). Then we examined if *β_v_* was dependent on the voxel's receptive field location as predicted by Equation (4).

To test for a genuine relation between β_*V*_ and vertical disparity, the test was repeated with the retinal disparity beta value (β_*R*_). Here, the voxel sub-group contained all voxels that showed significance (*p* < 0.05, uncorrected) on the polar angle mapping, and the eccentricity mapping, and on the GLM contrast of β_*R*_ against static baseline. Voxels' gain values (β_*V*_ or β_*R*_) were normalized with respect to each subject's peak response in V1 in order to compensate for general BOLD level differences between subjects.

For each voxel, the predicted RF location dependency was computed as explained above, and the Beta values (either β_*R*_ or β_*V*_) were obtained by application of the 4P-model on the each-subject's V1. For statistical testing and plotting, voxels were distributed in 10 equidistant bins, covering the range of the predicted RF location dependency value. Because the distribution of voxels' β_*V*_ and β_*R*_ response within each bin was highly skewed, the median was taken as a measure of central tendency, on which the linear regression analysis was applied.

### Model comparison

We assessed quality of fit of the 4P-model (Equation 2) on the fMRI data, by comparing it with the fit of the 8P-model (Equation 3). In the latter model, the amplitude of the BOLD response is fitted for each stimulus condition separately.

**Figure 7:**
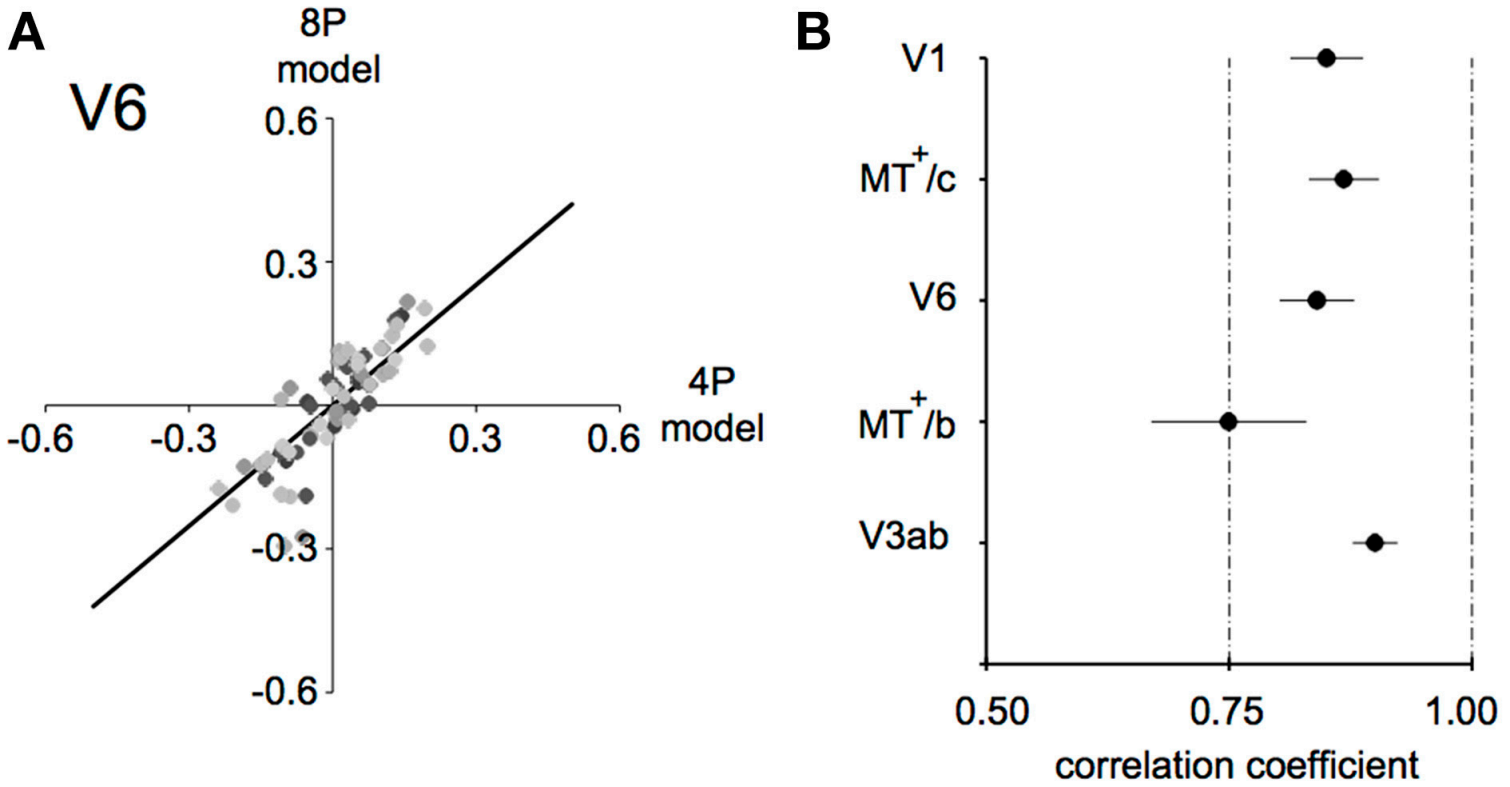
**Model fit comparison between the 4P-model and the 8P-model (A) illustration of the model fit for V6**. We started out by subtracting those components of the BOLD signal that explained the common response to the flow and low frequency drifts that was common to all conditions. Then, we computed the condition specific BOLD modulation explained by the binocular signals (4P-model) or by a hemodynamic function fit to each condition separately (8P-model). Thus, we arrive at two model fits for each condition/subject/ROI combination (black dots). The bold black line indicates the optimal linear fit of these points (least squares). **(B)** Average correlation values for each ROI between the 4P- and 8P-model. If the 4P-model would capture all of the BOLD modulations between conditions, its correlation with the 8P-model would reach 1.0, because the 8P-model serves as a reference of optimal fit of these modulations. Clearly the 8P-model achieves only a marginally better fit (±15%) in most ROIs and subjects.

For each subject, an amplitude estimate was obtained for both models. Next, these values were correlated for each ROI for each subject, as plotted in Figure 7A for area V6, and the average of the correlation value across subjects is plotted in Figure 7B.

For all subjects, comparisons of the beta values of the three confound predictors revealed no significant difference between the two models for any ROI [two-sided *t*-test, all ROIs *t*_(7)_ < 0.60, *p* > 0.56]. Hence, the confound predictors were excluded from the comparison, assuming equal estimates.

#### Inter-subject variability

To assess spatial congruence across subjects regarding V, R, and H sensitivity across cortex, we performed a Cortex-Based Alignment (CBA) analysis for both hemispheres. The inflated cortical representation of subject's left and right hemispheres separately were morphed into a spherical structure in which original curvature information of each vertex was maintained, which allowed for across subject analyses. Subsequently, all hemispheres were aligned by minimizing the curvature information between spheres, using the standard minimizing procedures of the CBA tool built-in in BrainVoyager QX (Goebel et al., 2006). The resulting curvature information of the averaged sphere, which also functioned as the target sphere for alignment, was used to reconstruct an inflated representation of left and right hemisphere. The single-subject GLM results of each subject, as depicted in Figures 4, 5, were then transformed to match the averaged hemisphere. This allowed us to establish for each contrast (V, R, and H) the proportion of overlap between the individual activation maps (at *p* < 0.05, uncorrected for each individual contrast), as plotted in **Figure 11**.

### Measuring vergence eye movements

Vergence eye movements were assessed in a separate session, in a dummy MR set-up, using an Eyelink II system (Eyelink® II, version 1.11, SR research, Canada). The experiment was performed in a dummy scanner with the same projector and very similar projection setup as during scanning. Contrary to the Eyelink® 2000 system available at the scanner, the Eyelink® II system uses two small separate cameras, which gave us the flexibility needed to measure eye movements in both eyes during stimulus presentation in the dummy scanner, with a screen close to the head.

Subjects (*n* = 5) wore contact lenses and underwent the same calibration procedure as in the main experiment, and lay in supine position in the dummy scanner bore. Subjects were instructed to attend to and look at the stereoscopic fixation ring at all times. Stimulus conditions were presented in exactly the same stimulus runs as in the main experiment, except for the static conditions, which were reduced to 4 s. Each subject completed four or five runs, and thus made 4 or 5 repetitions for each stimulus condition. Raw traces for left and right eye are illustrated for two conditions in **Figure 9B**, for one subject (S-1). Because the subject's eyes were very close to the projection screen, the Eyelink was calibrated only on a small portion of the actual screen and the Eyelink resolution was limited to about 0.4°, as can be observed in **Figure 9B**. Because sample frequency was high (500 Hz), gain and phase analysis of the low frequency vergence movement was hardly affected by these discrete steps (**Table 2**).

Saccades and blinks were removed from the left and right eye movement data and interpolated using a linear fit function. We ensured that the subject's eyes were positioned along the horizontal axis of the projection. Therefore, for each subject, and for each vergence condition separately, we defined the vergence signal as the difference of the left and right eye azimuth signal. For each subject, vergence signals were averaged over the 4/5 repetitions and fitted by a sine resulting in an amplitude and phase lags, as shown in **Table 2**. Horizontal drift values were assessed by fitting a linear function on the residuals of the sinusoidal fit (for the vergence conditions), or on the raw data after blink and saccade removal (for the non-vergence conditions).

The effect of inaccurate vergence eye movements on the GLM results were assessed by comparing the GLM results for a gain_1.0_ model (the original 4P-model with V_o_ and R_o_), a model with hypothetical gain values for each condition of 0.5 (gain_0.5_-model), and a gain_eye_ model. Here, V and R were adapted based on the measured gain in each condition (V_eye_ and R_eye_). Specifically:

$${\text{V}}_{\text{eye}}=\text{gain}{}^{*}\;{\text{V}}_{\text{target}}$$

and

$${\text{R}}_{\text{eye}}=\;|(\text{gain}{}^{*}\;{\text{V}}_{\text{target}}-\text{direction}{}^{*}\text{ H})|$$

Where direction refers to whether the direction of H is consistent (1) or opponent (−1) to the direction of V. Flow baseline and H are independent of V and thus are unchanged in the gain_eye_ model and gain_0.5_ model. For one subject with the lowest vergence gain, the described searchlight procedure was repeated for the gain_eye_ and gain_0.5_ model, and GLM contrast for R and H and RH were plotted on a flatmap representation of the cortex in both left and right hemisphere, using the same *p*-values as in the original model (**Figure 10**).

Goodness of fit (R^2^) for both models was examined by performing a single-subject ROI-GLM on each ROI for each subject in whom we measured pursuit in depth accuracy.

## Results

### Perception of depth modulation

All stimuli evoked a vivid percept of a rigid scene that approached and receded from the subject periodically. The magnitude of that percept varied across conditions in relation to the *binocular* components of the stimulus, because the optic flow as seen from the ego-center was identical. Subjects viewed multiple repetitions of all stimulus conditions in a random order, and rated for each stimulus condition the relative speed between themselves and the cloud of dots. Ratings were expressed as “slower” or “faster” relative to an internal mean, which subjects acquired over the course of the experiment.

Each stimulus condition is jointly characterized by its V, R, and H amplitude (Figure 3A). Which (combination) of these quantities matches the perceived motion in depth? Because the amplitude of V, R, and H were decoupled, one expects a monotonic (psychometric) relation between percentage slower scores and the level of those quantities contributing to the percept (Regan and Gray, 2009). We found, only for the level of H, a monotonic relation with the percentage slower (Figure 3B), indicating that the headcentric disparity amplitude modulated the perceived speed of motion in depth from flow.

### Localizers

In 8 subjects, we measured Blood-Oxygenated Level-Dependent (BOLD) signals using a Siemens Tim-Trio 3T scanner, using a previously described wide-field stimulus presentation (Arnoldussen et al., 2011). We performed a set of functional localizers to independently establish the location of motion responsive areas in dorsal visual cortex: our Regions of Interest (ROIs). These include V1, V3ab, and V6, which were identified using wide-field retinotopy (Sereno et al., 1995; Pitzalis et al., 2010), and also MT^+^-contralateral (MT^+^/c) and MT^+^-bilateral (MT^+^/b), by presentation of contra-lateral and ipsi-lateral flow (see also Materials and Methods). Average ROI Talairach coordinates are given in Table 1.

**Table 1:** **Mean Talairach coordinates of the ROIs in this study (± SD across subjects, *n* = 8)**.

**ROI**	**Left**			**Right**		
	***x***	***y***	***z***	***x***	***y***	***z***
V1	−10 ± 3	−77 ± 2	0 ± 4	8 ± 2	−74 ± 4	0 ± 5
V3ab	−17 ± 3	−87 ± 5	20 ± 8	18 ± 5	−86 ± 3	19 ± 4
MT^+^/c	−42 ± 2	−76 ± 4	7 ± 7	40 ± 5	−71 ± 4	7 ± 4
MT^+^/b	−44 ± 3	−69 ± 5	3 ± 5	44 ± 3	−64 ± 5	4 ± 5
V6	−13 ± 4	−76 ± 4	26 ± 5	14 ± 2	−75 ± 5	26 ± 5
VIP	−26 ± 3	−59 ± 8	46 ± 4	24 ± 5	−59 ± 6	45 ± 6

### Flow and binocular contributions to the BOLD signal

All subjects were exposed to multiple functional runs that contained all 8 experimental conditions, alternated by a baseline condition, presented in a blocked design.

We first established the contributions to the BOLD signal of the temporal modulations of the flow and the binocular signals. We used the 8P-model instead of the 4P-model to make minimal model assumptions. The V_0_R_0_H_0_ condition characterizes the BOLD response to binocular presentation of temporally modulated flow, without a *binocular* component to its modulation in time. Taking a grand average across subjects, across ROIs, we found a BOLD response to V_0_R_0_H_0_ of about 0.45% (V1: 0.41%, V3ab: 0.56%, MT^+^/c: 0.36%, MT^+^/b: 0.51%, V6: 0.43%).

Next, we examined the contribution of temporal modulation of the binocular signals to the BOLD signal, by performing in each ROI separately a multi-subject GLM contrast between the BOLD response to the V_0_R_0_H_o_ condition and the average of the remaining 7 conditions. The contrast (average of the 7 conditions > V_0_R_0_H_o_ condition) was significant for V1, MT^+^/b, V3ab, and V6 [mean modulation difference (mdf): 0.25%; 0.11%; 0.17%; 0.10%, two-sided *t*-test, *t*_(7)_ = 4.66; 2.53; 3.59; 3.29, *p* < 0.05], but not for MT^+^/c [mdf: 0.11%, *t*_(7)_ = 0.28, *p* = 0.78]. Hence, the addition of binocular self-motion cues to the flow stimulus evoked considerable modulations in the BOLD response, in all ROIs but MT^+^/c.

### Searchlight approach—individual subjects

To distinguish the different (V, R, H) contributions of binocular signals to the BOLD responses in flow sensitive areas, we used a searchlight approach, examining GLM contrasts (conjunctions of flow and any of the binocular components) from the 4P-model of individual data (Materials and Methods). For the *lateral* motion areas (MT^+^/c and MT^+^/b), we observed a strong R modulation that was localized within or at least encompassed MT^+^/b in 14 out of 16 hemispheres (Figure 4, flat map section); peak modulation by H was found less frequently (9 out of 14 hemispheres), and was most often located within MT^+^/c (7 hemispheres).

In most hemispheres, the R blobs were highly robust (*p*_min_ < 0.001 uncorrected in 11/16). The strong R modulation is illustrated by the response preference for the stimulus with the highest speed of retinal disparity (V_4_R_6_H_2_) rather than headcentric disparity (V_4_R_2_H_6_) in R-sensitive regions when vergence was identical (Figure 4, graphs).

For the *medial* motion areas, we observed strong modulation by H in all subjects (V3ab, V6, Figure 5, flat map section). H modulation peaks were found in all hemispheres (16/16) in V3ab. Also in V6 (7/16 hemispheres) such peaks were found. In some subjects the contrast in H regions between responses to V_4_R_2_H_6_ and V_4_R_6_H_2_, showed a clear preference for the former (Figure 5, graphs), but in most subjects the contrast was less pronounced because of co-modulation of that ROI by R. Half of the hemispheres (8/16) showed also a peak R modulation in V3ab; one hemisphere showed peak R modulation in V6.

The searchlight approach suggested a stronger response to H compared to R within medial motion-responsive cortex. In contrast, a sub-region of MT^+^/b responded more specifically to R. To find out whether the whole ROI had a response bias to one or the other type of binocular signal, we performed a random effects multi-subject GLM, for each ROI separately, using the 4P-model. Now, all voxels of the ROI were included without selection of voxels that responded significantly to flow. Congruent with the individual patterns of activation, MT^+^/c, V3ab and V6 were significantly modulated by H [Figure 6A, two-sided *t*-test: MT^+^/c: *t*_(7)_ = 3.52, *p* < 0.01; V3ab: *t*_(7)_ = 5.08, *p* < 0.01; V6: *t*_(7)_ = 7.15, *p* < 0.001]. H modulation was significantly larger than R modulation in V6 and V3ab [*t*_(7)_ = 3.15; 3.41, *p* < 0.05]. V1 and MT^+^/b were significantly modulated by R [*t*_(7)_ = 4.25; 4.46, *p* < 0.01].

The use of a VM torus generates changes in disparity not captured by the 4P-model that are notable for eye elevation angles larger than 25° (see Materials and Methods). To exclude possible confounding effects by these changes to the reported signal changes by V, R, and H, we repeated the multi-subject analysis, using only those voxels of which the preferred response was located within 25° of elevation in the visual field (calculated using the results of retinotopic mapping, see Materials and Methods). For all regions, this resulted in the exclusion of maximally 13 percent of voxels on average across subjects (for area V6). The results are very similar to the original analysis (Figure 6B). MT^+^/c, V3ab and V6 were significantly modulated by H [two-sided *t-test:* MT^+^/c: *t*_(7)_ = 3.43, *p* < 0.05; V3ab: *t*_(7)_ = 7.79, *p* < 0.001; V6: *t*_(7)_ = 7.79, *p* < 0.001]. H modulation was significantly larger than R modulation in V6 and V3ab [*t*_(7)_ = 3.01; 3.01, *p* < 0.05]. V1 and MT^+^/b were significantly modulated by R [*t*_(7)_ = 4.13; 4.51, *p* < 0.01].

In sum, we found a notable difference in sensitivity to binocular signals in medial motion areas (high H sensitivity) and lateral motion areas (high R sensitivity).

### Vergence response in area V1

**Figure 8:**
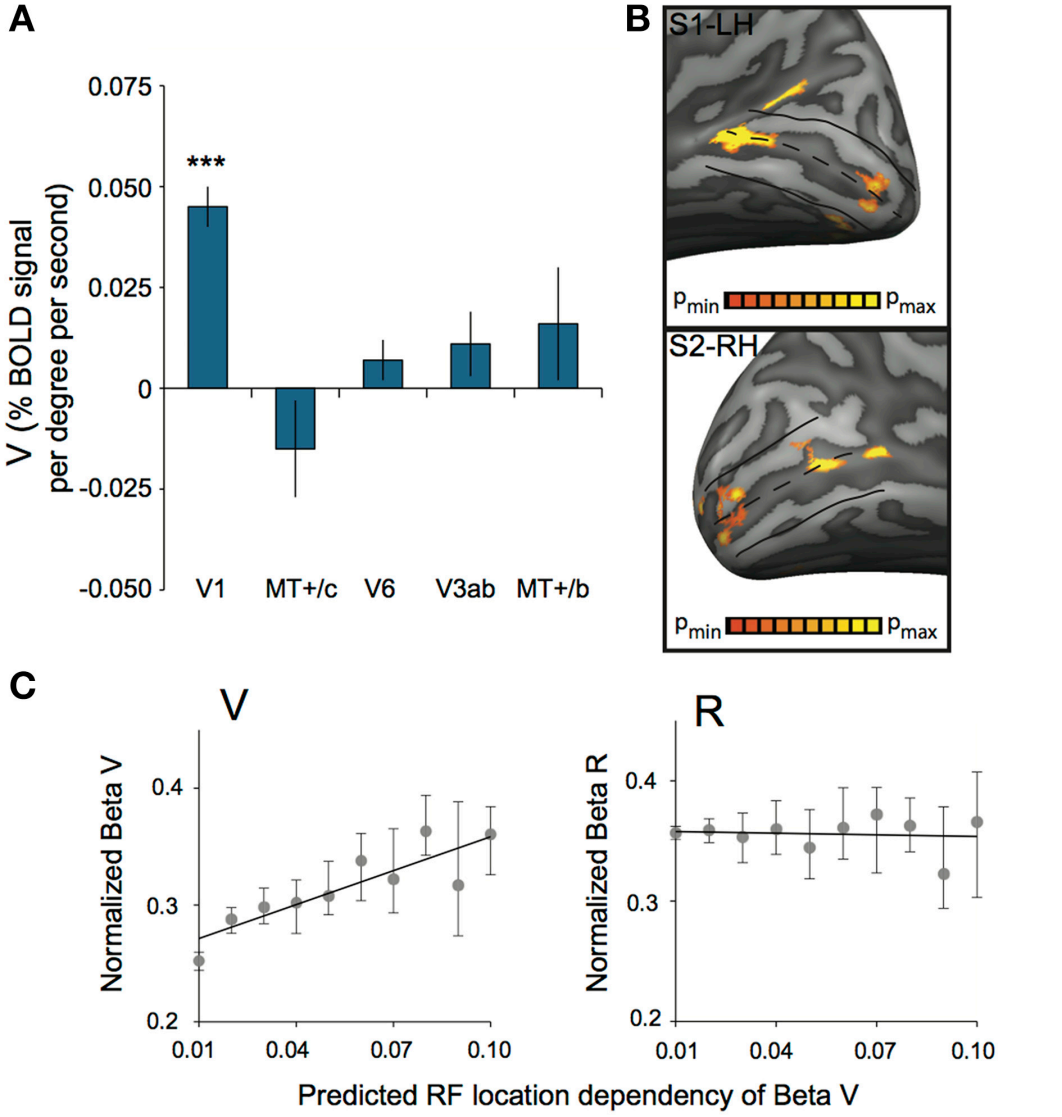
**Vergence (V) response in striate cortex. (A)** Sensitivity to V across subjects for each ROI. Only V1 was modulated strongly by V (^***^p < 0.001). **(B)** V activation plotted on left and right hemisphere of two different subjects, at high significance threshold (*p* < 0.00001 uncorrected). A clear non-uniform distribution of V sensitivity is observed, resulting in one more foveal and one more eccentric activation blob. **(C)** Normalized beta plot of median voxel sensitivity to V (β_*v*_, left graph) or R (β_*r*_, right graph), ordered by their predicted RF location dependency (tan(α) ^*^ sin(2θ); binned in 10 equi-distant groups). Error bars represent 95% confidence intervals of the median for each bin, obtained by a bootstrap procedure (1000 samples per bin). The black line in both graphs illustrates the linear model fit. There is a significant linear relation between predicted RF location dependency and the β_*v*_ for V-responsive voxels (Model *R*_square_ = 0.76, *p* = 0.001), but not for R-responsive voxels (Model *R*_square_ = 0.01, *p* = 0.78).

Additionally, we assessed ROI responsiveness to vergence eye movements (V) across subjects, using the multi-subject GLM. We found significant activation to vergence eye movements only in V1 [*t*_(7)_ = 8.86, *p* < 0.0001, other ROIs: *t*_(7)_ < 1.36, *p* > 0.21, Figure 8A]. Inspection of the distribution of V activation across V1 showed that vergence response was distributed unevenly across the cortical surface. In most subjects, an anterior and a posterior region with strong modulation by eye vergence could be distinguished (Figure 8B). Either sub-region's response could be due to an efference copy signal or due to a *visual* correlate of eye vergence.

An efference copy signal of eye vergence should not depend on the visual direction. For vertical disparity however, we know that it depends on horizontal vergence *and* cyclopean visual direction (Read et al., 2009). Thus, we looked in retinotopic area V1 for a visual direction dependent magnitude of the vergence response (as characterized by the GLM's Vergence Beta value: β_*V*_). (see Materials and Methods).

To test this prediction, we selected for each subject, all significant voxels in area V1, both for the retinotopic localization fits (polar angle and eccentricity mapping) and β_*V*_ (*p* < 0.05 on each test). We limited the search to an eccentricity of 45°; resulting in a grand total across subjects of 3928 voxels that passed all criteria. Subsequently, we computed for each voxel the magnitude of the local vertical disparity level. This was done based on its RF location within the visual field, and the geometric relations between vertical- disparity and horizontal vergence (see Materials and Methods).

We established a gain factor *g* = 0.83 and a constant *C* = 0.27 from the regression (Figure 8C, left graph), indicating a contribution of vertical-size disparity to the vergence response in area V1 and possibly a non-visual contribution.

We applied the same procedure to the voxels in area V1, but now with significant values of β_*R*_ and retinotopic localization fits (Figure 8C, right graph). This analysis involved about 5% more voxels (4164), but yielded no significant relation with the predicted function for vertical disparity (*g* = −0.05, *C* = 0.36).

Finally, to assess potential effects of the non-ideal control of horizontal retinal disparity at large elevations, we repeated the above analysis for each subject selecting only those voxels with a preferred stimulus location within ±25° elevation. This selection removed for each subject less than 3% of the voxels. This had marginal effects (in the 3rd decimal) on the slopes and offsets reported above.

### Model quality on capture of parametric modulations

We explain the differential responses to the different stimulus conditions by three binocular parametric predictors (β_1_^*^ *V_i_* + β_2_^*^ *R_i_*+ β_3_^*^ *H_i_*) on top of the flow response (β_4_^*^ Fb), which is common to all conditions (4P-model). How successful is this model compared to a model where each condition is fitted separately by a BOLD response template (8P-model)? To find out we subtracted from each model fit the common flow component from the 4P-model:

(5) $$R_{4P,i}=\eta_{1}{}^{*}V_{i}+\beta_{2}{}^{*}R_{i}+\beta_{3}{}^{*}H_{i}(i\in [1,8])$$

(6) $$R_{8P,i}\;=\beta_{i}-\beta_{4}{}^{*}Fb(i,\in [1,8])$$

In each ROI, we regressed the model predictions R_4p_ and R_8p_ against each other, for all subjects and conditions, as illustrated in Figure 7A for area V6. The β's of the 4-parameter model captured the vast majority of the BOLD modulation over stimulus conditions (Pearson's correlation: lowest value: 0.74 in MT^+^/c, Figure 7B). This indicates that in our target ROIs a linear response to the amplitude of vergence, retinocentric- and headcentric disparity modulation explains 75–90% of the hemodynamic response on top of the BOLD response to flow.

### Measuring vergence eye movements

For the 4P-GLM-model, we based the scaling of the parametric predictors on the stimulus amplitude of the binocular components. The scaling assumes perfect fixation on the target ring that moved in depth. Hence, it is key to the interpretation of the BOLD signals that subjects make accurate vergence eye movements during all eye vergence conditions. Unfortunately, the wide-field projection system precluded measurement of eye movements in the scanner. In a separate session in a dummy MRI-setup, we assessed the accuracy of eye vergence movements for each stimulus condition using an Eyelink II system (Eyelink® II, version 1.11, SR research, Canada). Gain and phase lag for all 6 vergence stimulus conditions were assessed in five of the subjects that had participated in scanning. We also assessed linear horizontal drift during each trial.

**Figure 9:**
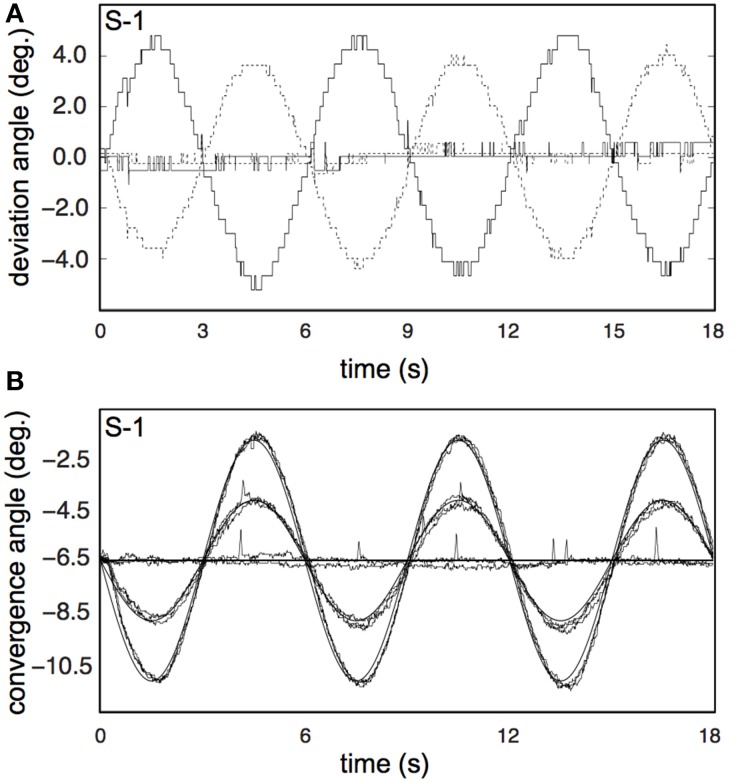
**Results for the vergence eye movement recordings. (A)** Raw traces of eye pursuit in depth measurements from subject S-1, for one of the high vergence conditions (V_4_R_0_H_4_, bold lines), and one of the non-vergence conditions (V_0_R_0_H_0_, normal lines). Dotted lines represent left eye traces; solid lines represent right eye traces. The apparent low sample frequency is due to the manual calibration of the Eyelink system (see Materials and Methods). **(B)** Pursuit eye movements in depth from S-1, for all 8 conditions. Each trace shows the average of all repetitions for that condition, after blink removal and linear drift correction. Bold lines represents fixation movement in depth for *V* = 2 and *V* = 4.

Subjects' vergence eye movements showed minimal phase lag, nearly perfect vergence pursuit for all stimulus conditions (Table 2, Figure 9 for a single subject). Horizontal drift during each trial was minimal and likely reflects drifts of the head relative to the camera assembly rather than real eye movements (Table 2).

**Table 2:** **Gain and phase lag of eye vergence averaged across subjects (*n* = 5)**.

	**Gain (deg)**	**Phase (deg)**	**Drift (deg)**	**Gain S8**
V2R2H0	1.01 (0.02)	4.62 (1.71)	−0.30 (0.07)	0.96
V2R4H2	1.01 (0.02)	3.50 (0.69)	0.21 (0.36)	0.96
V2R0H2	1.01 (0.02)	5.17 (1.22)	−0.21 (0.07)	1.03
V4R0H4	1.00 (0.02)	5.56 (0.51)	−0.06 (0.05)	0.94
V4R2H6	1.00 (0.02)	5.48 (0.82)	0.07 (0.20)	0.99
V4R6H2	0.94 (0.05)	6.64 (1.96)	0.07 (0.18)	0.90
V0R0H0			0.06 (0.17)	
V0R4H4			0.02 (0.08)	

*Gain and phase lag are shown for each of the 6 vergence conditions, and average horizontal drift is shown for all 8 stimulus conditions. Gain is expressed as the ratio of the vergence amplitude to the fixation target vergence amplitude. Positive phase difference indicates a lag of the eye vergence position over time compared to the position of the fixation point over time. The horizontal drift expresses the total drift in degrees over the course of a trial. The final column shows the gain values for subject 8. SEM is given in parentheses*.

**Figure 10:**
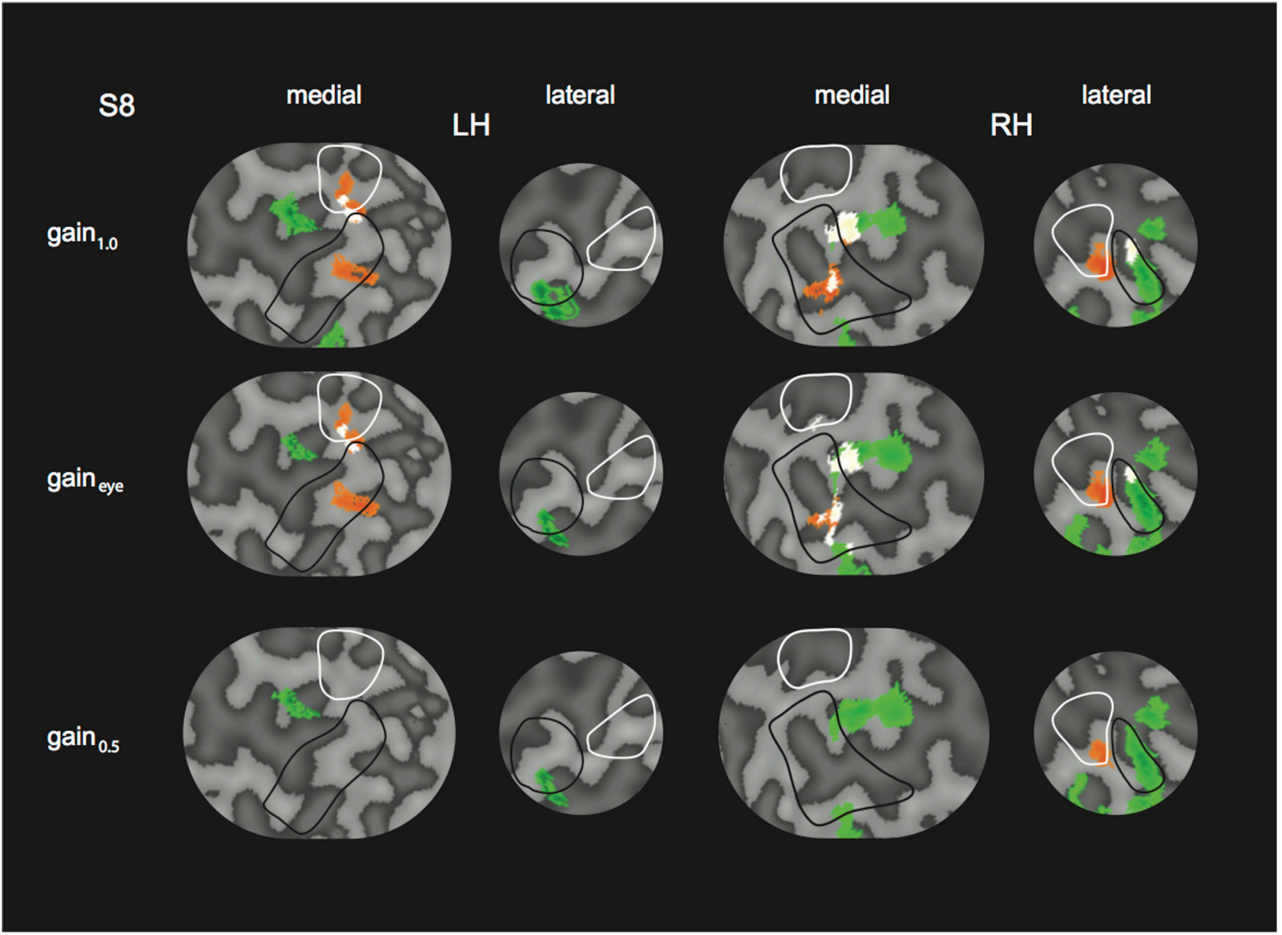
**Modulation by binocular signals for the 4P-model, with predictors adapted for different gain levels**. Shown are sections of the flattened representation of both hemispheres in subject 8, encompassing medial and lateral motion areas. On top, peak activations are drawn for R (green—dark green), H (orange—red), and RH (white—yellow), for the gain_1.0_ model, the gain_eye_ model, and the gain_0.5_ model. P(min) was set to the same level as in the original model (Figures 4, 5). The gain_eye_ model was adjusted using measured gain values in subject 8 (V2R2H0: 0.96; V2R4H2: 0.96; V2R0H2: 1.03; V4R0H4: 0.94; V4R2H6: 0.99; V4R6H2: 0.90).

We tested the influence of inaccurate vergence eye movements on the GLM results, by changing the GLM predictor values from the original model (gain_1.0_ model, i.e., 4P-model) based on the gain values obtained from eye tracking (gain_eye_ model). We did so, for the subject with the lowest gain values (S8, gain values in Table 2). Changes in R and H modulation showed minimal changes on the flat maps (Figure 10). We also assessed the influence of inaccurate gain by changing the GLM predictors based on a hypothetical gain of 0.5 (gain_0.5_ model). Here, R and H activity changes were clearly visible on the flap maps (Figure 10). For all subjects, we examined if the measured-gain model outperformed the perfect gain-model, by comparing goodness of fit (R^2^). Across subjects for each ROI, *R*^2^-values did not differ significantly [paired two-sided *t*-test, *t*_(4)_ = 0.50; 1.17; 1.00; 1.05; 1.04, for MT, MST, V6, V3ab, and V1 respectively].

Overall, the similar results for the gain_eye_ model show that the observed vergence eye movement inaccuracies do not affect our conclusions.

### Inter-subject variability

The search light approach identifies locations with peak H,V or R sensitivity in different subjects. Although the ROI analysis confirms the separate sensitivity to H, V, and R tuning at the group level (4P-model, Figure 6) in different ROIs, it is of limited use for inferring spatial congruence between the subjects regarding loci with H or predominant R sensitivity across dorsal and ventral cortex. To examine spatial congruence, we projected the functional maps of each subject onto an averaged inflated representation of left and right hemisphere, which was computed using a Cortical-Based Alignment (CBA) analysis. CBA analysis projects single subject hemisphere curvature information on a common spherical representation and subsequently minimizes curvature differences across subjects, resulting in an alignment based on cortical curvature information (Goebel et al., 2006).

**Figure 11:**
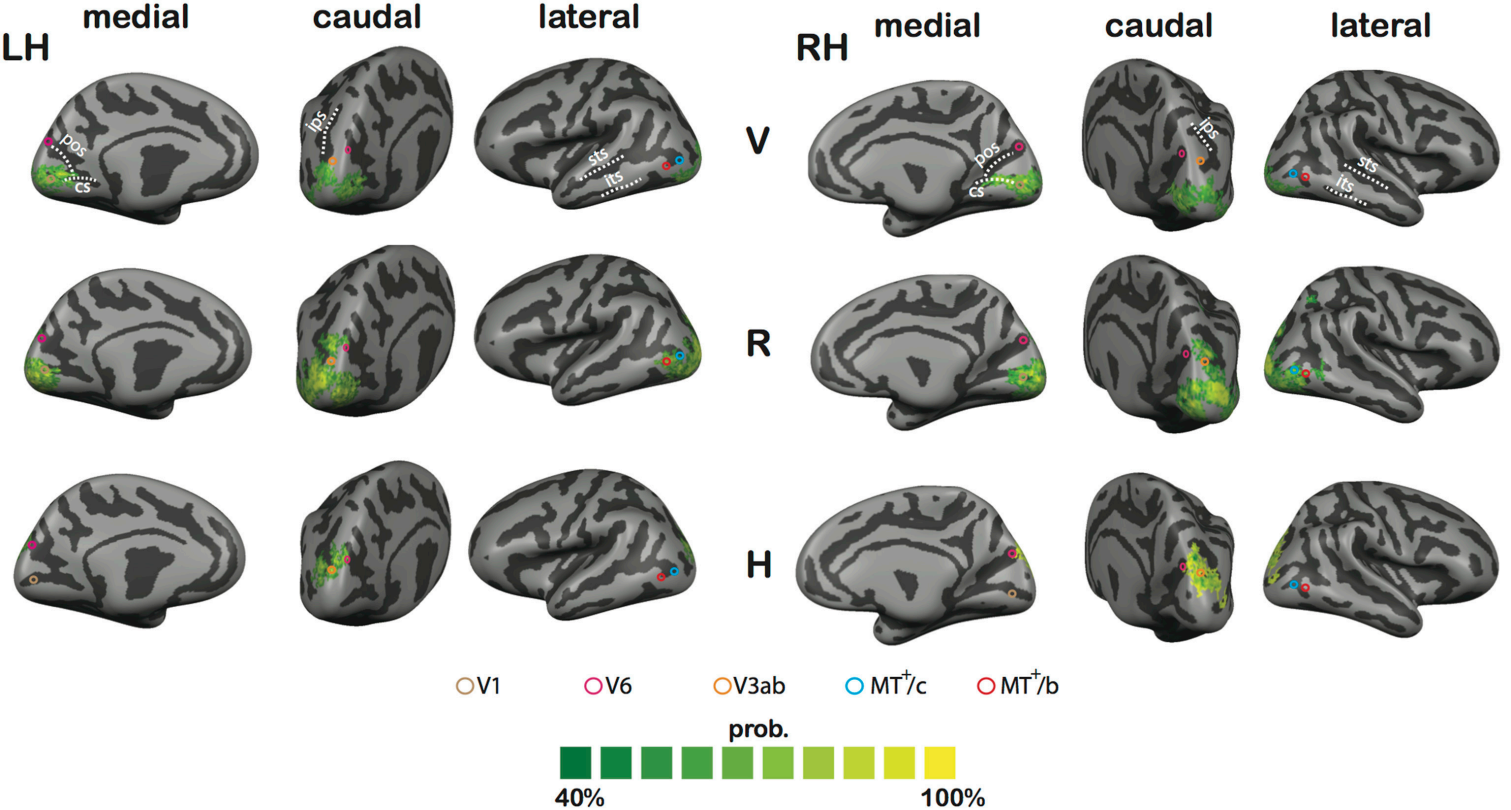
**Results of the CBA analysis**. Shown is the proportional spatial overlap (green to yellow) between subject's functional maps for V (top tow), R (middle row), and H (bottom row; *p* < 0.05, uncorrected for all individual contrasts). The proportion is projected on the averaged representation of left (LH) and right hemisphere (RH), which was derived from the average of all curvature information after CBA across subjects. Circles define approximate ROI locations, based on the Talairach coordinates reported in Table 1.

As a result, each subject's functional maps could be transformed to a singular target sphere, which consisted of an average of all 8 hemispheres. For V, R, and H separately, we assessed spatial congruence by computing the proportion of subjects that showed significance for the contrast for each vertex (*p* < 0.05, uncorrected, Figure 11). The highest proportion of V activation was found in V1, along the calcarine sulcus. A high proportion of R activation was found along both dorsal and ventral stream, covering all our ROIs. This seems in contrast with the results of the multi-subject analyses, where R did not reach significance in MT^+^/c, V3ab, And V6, pointing to weak but consistent R sensitivity in these areas. Among our ROIs, the highest proportion of H activation is restricted to the more foveal part of V6, while V3ab seems fully covered. The results also uncover an additional region of consistent H activation that falls outside our pre-defined ROI, which likely corresponds to putative area V7. Regarding R activation, a large consistent region of activation is seen lateral and posterior to V3ab. This region most likely coincides with the Lateral Occipital (LO) region, based on anatomical location (Larsson and Heeger, 2006), and previously reported sensitivity to changing retinal disparity (Rokers et al., 2009).

In sum, the CBA results further bolster the observation that H activation is confined to Posterior Parietal Cortex and Posterior Occipital Cortex. Lower R thresholds reveal consistent (but probably weaker) R activation along both lateral and medial motion areas.

## Discussion

Monocular optic flow provides important information for the direction of heading (Lappe et al., 1999). Many believe *binocular* information is superfluous. Yet, heading judgment can operate on purely stereoscopic cues (Macuga et al., 2006). Judgment of the speed of self-displacement in depth may profit even more from binocular signals than perception of self-motion direction; the inherently temporal information from optic flow (Koenderink, 1986) does not provide speed. It needs extension with a spatial scale that may be mediated through binocular information. Our observation that perceived speed of sinusoidal back-and-forth self-motion is enhanced by concurrent sinusoidal headcentric disparity, suggests that binocular signals may contribute to such a headcentric distance scale to the flow. Because retinal disparity refers to depth relative to the fixation point, modulation by R indicates an influence of speed by which the scene approaches the fixation point. In contrast, modulation by H indicates a true influence of the approach speed between the head and the scene. It remains to be seen, however, whether more veridical distance judgments arise from the combination of optic flow and headcentric disparity. Our observation extends previous psychophysical reports that speed judgments of approaching objects include changing eye vergence information and changing relative retinal disparity (Howard, 2007; Lugtigheid et al., 2011). These studies did not distinguish between contributions of headcentric disparity and eye vergence as we do here.

Our observation bolsters an earlier observation (Arnoldussen et al., 2013b) that perceived self-rotation depends on the rotational flow relative to the head and not relative to the gaze line. That study and the present indicate that the visual flow relative to the *head* is paramount to the self-motion percept. The visual system, extended with eye movement signals, appears to gather information about the rotation and translation of the *head* relative to the scene. This shift from visual retinal flow to headcentric flow was proposed before (Beintema and Van den Berg, 1998; Arnoldussen et al., 2013b) as a step to align *visual information on* self-motion with the information from the *vestibular* system, which reports the head's motion relative to the world.

It is known that visual and vestibular information combine optimally for perceived heading direction (Gu et al., 2008), but so far comparable data for visuo-vestibular judgment of speed (translational or rotational) of self-motion are lacking as far as we know.

Only areas V3ab and V6 were modulated strongly by amplitude changes in H. In contrast, the BOLD modulation in MT^+^/b showed strong modulations to R, but none to H (Figures 4, 5). This distinction between V3ab/V6 and MST^+^/b with regard to the type of binocular sensitivity provides strong support that analysis of motion signals in the dorso-medial and dorso-lateral pathways (Galletti and Fattori, 2003; Kravitz et al., 2011; Pitzalis et al., 2012b) serve different functions.

### Dorso-medial responses

Human V6 shows strong BOLD responses to full field optic flow (Pitzalis et al., 2010), specifically patterns that contain depth by motion parallax (Pitzalis et al., 2013). Also, V6 integrates depth from motion parallax with depth by binocular disparity (Cardin and Smith, 2011), possibly to rescue direction of heading perception from contamination by motion noise, and to distinguish the depth components of multiple moving objects (Arnoldussen et al., 2013a). These studies did not distinguish between retinal and headcentric disparity.

Headcentric disparity (H), more than retinal disparity is suited for the integration with motion parallax, because it shares with parallax a dependency on target distance to the head, whereas retinal disparity depends on distance to the fixation point. Thus, the strong modulation of optic flow responses by H suggests an important contribution of V3A/V6 to process the *speed* of the head's movement in depth. This lines up nicely with a previous study concluding that V6 combines retinal rotational motion and eye *pursuit* signals to indicate the speed of rotation of the head in space rather than rotation speed of the gaze line (Arnoldussen et al., 2011, 2013b).

Other findings suggest that the flow-sensitivity of human V6 may not point to a role in perception of the *direction* of self-motion; fMRI adaptation in V6 does not rise for repeated presentation of the same focus of expansion of optic flow (Cardin et al., 2012). Also, one might expect sensitivity to the vestibular modality to perceive the direction of heading correctly but this appears to be lacking in area V6 (Smith et al., 2012). Because these studies presented monocular flow one cannot exclude processing of the direction of heading for binocular stimuli for now.

Further functional considerations follow from the comparison with Macaque V6. The majority of V6 neurons are strongly motion-sensitive (Galletti et al., 1996, 1999), but its response to optic flow remains unstudied. Macaque V6 lies within the parieto-occipital stream and has strong connections to parietal area V6A (Galletti et al., 2001), an area strongly related to reaching (Fattori et al., 2001, 2005). Many neurons in V6 are “real-motion cells,” meaning they distinguish “real” retinal motion from eye-movement evoked retinal motion (Galletti and Fattori, 2003). These findings led to the idea that V6 plays a role in visually guided actions (Pitzalis et al., 2012a). Our findings support this notion, because the processing of the speed of head rotation (Arnoldussen et al., 2011) and head translations (current study) from optic flow would serve proper control of motor actions, especially in peri-personal space, such as reaching, grasping, and repelling objects that approach the head.

The virtually lacking modulation by headcentric disparity in the MT^+^/b complex (Figure 4) suggests that the speed of the head's movement in depth is not processed there. What might be the reason for the strong modulation by retinocentric disparity? This region shares certain properties with activity evoked in the medial temporal cortex, area MST, of the monkey: sensitivity to self-motion specific patterns of optic flow in contra and ipsi-lateral parts of the visual field. Such patterns provide direction of self-motion and depth information but the informative component must be separated from the uninformative rotational component of the flow, which does not carry depth information (Warren and Hannon, 1988). To support that separation, primate self-motion perception relies on non-visual information, like efference copy signals of eye movements (for reviews: Lappe et al., 1999; Britten, 2008) and vestibular signals of head movements in MST (Angelaki et al., 2009, 2011). It also uses independent signals on depth order (Van den Berg and Brenner, 1994b). Depth order can be derived from many sources including both types of binocular disparities. Perhaps retinal disparities are preferred to establish depth order as they are less computationally expensive.

### Vergence eye movement modulation in V1?

The ocular convergence angle required for fixating an object, signals a target's distance relative to the head (Von Hofsten, 1976; Foley, 1980; Viguier et al., 2001). Neuro-physiological work points to the integration of the eye vergence signal with retinal disparity for reach distances in various regions within PPC, for example the parietal reach region (PRR; Bhattacharyya et al., 2009). Early neurophysiological work reported modulation of disparity-selective neurons by eye position signals as early as V1 (Trotter et al., 1992, 1996). Also V1, V2, and V4 neurons' responses to a contrast target are up regulated by increasing eye vergence angle (Rosenbluth and Allman, 2002). However, both studies have been criticized on methodological grounds (Cumming and DeAngelis, 2001).

Here, we show strong modulation of the BOLD signal by V and R in area V1 (Figures 6, 8A). Geometrically, changes in retinal *vertical* disparity correlate with eye vergence, and depend only on visual direction. Therefore, *changes* in V and *changes* in vertical disparity take place concomitantly. Interestingly, that visual direction dependency was reflected in the magnitude of the BOLD response to vergence within area V1 (Figure 8C, left graph), but not at all in the response to the amplitude of the horizontal retinal disparity in V1 (Figure 8C, right graph). Vertical disparity alternates polarity between visual field quadrants, but polarity is unlikely to be represented in the BOLD signal. Hence, the nature of the BOLD signal prevents us to establish the presence of that polarity change and we cannot distinguish a response to vertical disparity from the mere vertical-size mismatch of the image. Nevertheless, our data strongly suggest that the modulation by V in cortical area V1 at least reflects sensitivity to vertical-size disparity. To our knowledge, this is the first human evidence for vertical-size disparity sensitivity in V1, already demonstrated in Macaque striate cortex (Cumming, 2002). It appears then that V1 may represent eye vergence from the global pattern of vertical size mismatch between the eyes' images.

### Caveats—vection

Unequal attentional load between stimulus conditions can alter the BOLD modulations (Huk and Heeger, 2000), also in MT^+^/b and MT^+^/c (Treue and Maunsell, 1999). To prevent differences in attentional load due to vection, we instructed subjects to keep attention on the fixation point in depth and to make vergence eye movements as accurately as possible. Also, peak forward speed of the simulated motion was slow (300 mm/s), and *post-hoc* reports by the subjects indicated no strong percepts of vection (not quantified). Importantly, the optic flow was similar for all stimulus conditions. Because the optic flow remains the primary and strongest visual cue to self-motion, irrespective of additional binocular cues, vection differences between stimulus conditions were minimized. Overall, the effects described are unlikely due to attention or vection.

### Caveats—independent control of retinal and headcentric disparity

Our study relies on independent control of retinal headcentric disparity and eye vergence. As we mentioned before, geometric properties of the disparity field preclude a full dissociation of retinal horizontal disparity and headcentric disparity across the VM surface. We showed by simulations that changes in retinal horizontal disparity associated with the changes of the position of the virtual screen (VM surface) and the fixation point in depth were very close to the intended values and even better so when the field of view was limited to the central range (±25°) of elevation. We found that only for a small minority (<13%)of the voxels in our ROIs the receptive field (RF) was located at elevations beyond 25°. Accordingly, we found little change in the results (Figures 6A,B) when we limited the analysis of the 4P-model to the voxels with RFs within the central ±25° of elevation. Also, we note that when we entertain the hypothesis that voxels responsive to headcentric disparity would actually respond to spurious horizontal retinal disparities we should be puzzled why such voxels are not found in area V1? Hence, we conclude that our choice of the VM surface for the virtual screen was appropriate to distinguish headcentric and retinal disparity tuning in the ROIs.

### Caveats—vergence eye movements

Can inaccurate vergence eye movements explain our results? The wide-field projection system did not allow for online registration of eye movements. Vergence eye movement accuracy for the different stimulus conditions was measured in 5 of the subjects in dummy scanner setup. Subjects made accurate vergence eye movements for all stimulus conditions (Figure 9, Table 2), with gain and phase values comparable to previously published data (Erkelens and Collewijn, 1985a).

An adaptation of the 4P-model that reflected adapted V and R predictor values given non-perfect gain (gain_eye_ model) showed minimal differences in the single subject results compared to the original model (Figure 10) Across subjects, the gain_eye_ model did not improve on the original model. We conclude that inaccurate vergence eye movements cannot explain our fMRI results.

### Caveats—relative retinal disparity

We note that our projection method on the virtual screen removed *relative* retinal disparity associated with the positions within the cloud of individual dots. Therefore, the changing relative retinal disparity normally associated with self-motion through a 3D cloud was not presented. We cannot rule out the possibility that inclusion of relative disparity information would contribute to the percept motion in depth, as has been found experimentally (Erkelens and Collewijn, 1985b; Brenner et al., 1996; Welchman et al., 2009). In light of this work, our results show at least that by minimizing relative disparity information, the contribution of other binocular cues to perceived motion in depth is revealed. Two other reasons make us doubt a strong contribution of relative disparities to the percept of motion depth. First, we note that headcentric disparity, absolute retinal disparity, and relative retinal disparity provide decreasing headcentric information in that order. Secondly, relative retinal disparity *was* present between the fixation point and the dots, in all conditions in which the binocular fixation point was not placed on the virtual projection screen (i.e., *R* > 0). We did not find evidence that these conditions provided especially large BOLD signals. Nevertheless, to clarify, a potential contribution of relative disparity signals to the perceived speed of self-motion needs further investigation within the context of our type of stimuli.

### Caveats—cyclovergence and tilted horopter

Our stimulus presentation did not take into account cyclovergence of the eyes. Cyclovergence that is coupled to horizontal vergence occurs when the primary position is not located in the plane of regard. We did an analysis of the effect of adding elevation dependent cyclovergence (Mok et al., 1992; Van Rijn and Van den Berg, 1993) to the model, because we did not know the primary position of our subjects' Listing planes. We varied the elevation of the assumed primary position by 10°, which then results in cyclovergence coupled to the imposed horizontal vergence, and associated vertical disparities (using full equation 1 from Read et al., 2009). We found only marginal changes of fit of the linear regression by the inclusion of that cyclovergence component (not shown). Hence, our data suggest that the tilt of the eyes in opposite directions has not been an important contribution to the BetaV fits of Figure 8C.

The horopter shows a backward tilt in the vertical dimension (von Helmholtz, 1962). We did not take into account this tilt, because our hypothesis involves a change of the vertical size disparity over time. This would not be affected by an offset like the tilt of the horopter.

### Conflict of interest statement

The authors declare that the research was conducted in the absence of any commercial or financial relationships that could be construed as a potential conflict of interest.
