# Supplementary material for: Dissociation of retinal and headcentric disparity signals in dorsal human cortex
Source: Front Syst Neurosci. 2015 Feb 24;9:16. doi: 10.3389/fnsys.2015.00016 (PMC4338660; doi:10.3389/fnsys.2015.00016)
Supplement: Supplementary file 1 [file DataSheet1.DOCX]

# Supplementary Material – Methods

**Vertical size disparity - Rationale.** We observed in V1 a strong BOLD modulation by V, which was distributed unequally across the cortical surface (Figure 8b). We hypothesized that this activation is caused by the changing vertical retinal disparity during vergence eye movements. Vertical disparity depends on visual direction (Helmholtz angles azimuth (α) and elevation (θ)) and the convergence of the eyes (TV of the fixation point: V_f_). This is captured by the following formula:

(4)

This formula is based on Equation 1 of Read et al (2009) with the constraints that the mean and difference of the eyes’ torsions, horizontal cyclopean eye orientation and the vertical vergence are zero (Read JC et al. 2009). This is appropriate for the experiments that we conducted because subjects only made slow vergence eye movement along a trajectory straight ahead at eye height.

The cyclopean direction of each voxel was computed using each voxel’s polar angle (μ) and eccentricity (ecc) fit from retinotopic mapping. The term tan(α) is given by:

(5)

The term 0.5sin(2θ) is given by :

(6)

where:

(7)

Thus, the BOLD activation component in each voxel of area V1 (BOLD_V_) that is attributed to vergence (β_V_),

(8)

could also derive from binocular units that respond to vertical disparity:

(9)

If so, one would predict the measured β_v_ from the GLM model to depend on the receptive field (RF) location or visual direction of the voxel with a certain gain factor (g):

(10)

In contrast, for a pure efference copy related response that does not depend on the visual direction one would predict: β_V_ = Co.

Each voxel’s predicted linear relation between the normalized β_V_ and the vertical disparity at each retinotopic location is as follows:

## (11)

where C and g are factors of the linear regression. The constant factor (Co) allows for the possibility of a non-retinal component of the vergence signal in area V1.

The global pattern of vertical disparity across visual field scales with vergence, and changes polarity from one quadrant to another (figure 7 Read et al.). However, whereas the scaling is represented by modulation in BOLD amplitude, information about polarity cannot be deduced. A linear relation between β_V_ and the vertical disparity therefore shows a global sensitivity to the vertical size difference between left and right image (vertical-size disparity). We explicate this by referring to vertical-*size* disparity rather than vertical disparity.

**Further stimulus considerations regarding intended and actual disparity amplitudes.**

As mentioned above our choice of the VM surface allowed for approximately independent control of the amplitudes of the horizontal component of retinal disparity and head centric disparity. To estimate the deviations that could occur in our wide-field presentations we computed the amplitudes of the horizontal-retinal disparity averaged across the display dimensions (azimuth range ±60 deg; elevation range ± 45 degrees). We simulated pure horizontal vergence along the intersection of the horizontal and the mid-sagittal plane, without cyclovergence. Interocular distance was 65 mm. We simulated the most extreme stimulus excursions i.e. headcentric disparity with 6 deg amplitude (V4R2H6) and retinal disparity with 6 deg amplitude (V4R6H2). The headcentric disparity accorded with the nominal values (6 and 2 degrees) because it is by definition independent of the eye orientation, and because the head was stabilized. Numerical analysis showed that – when *averaged across the elevations beyond 25 deg* - retinal disparity of 2.75 deg amplitude occurred in the V4R2H6 condition. But limiting the field to ±25 deg elevation, an amplitude of 2.15 deg was found for V4R2H6 with an intended retinal amplitude of 2 degrees.

The sinusoidal *retinal* disparity stimulus of 6 deg amplitude (V4R6H2 condition) actually evoked a -5.6 to + 6.1 deg excursion of retinal disparity within the field of ±25 deg elevation.

Thus, for elevations lower than 25 deg the deviations in the amplitude of retinal disparity were on average less than 10% of the intended amplitudes.

## 
